# What corpus data reveal about the Position of Antecedent Strategy: anaphora resolution in Spanish monolinguals and L1 English-L2 Spanish bilinguals

**DOI:** 10.3389/fpsyg.2023.1246710

**Published:** 2023-11-09

**Authors:** Cristóbal Lozano, Teresa Quesada

**Affiliations:** Universidad de Granada, Granada, Spain

**Keywords:** Spanish second language acquisition, anaphora resolution, position of antecedent strategy, learner corpora, pronominal subjects, CEDEL2 corpus

## Abstract

This study investigates the acquisition of anaphora resolution (AR) in Spanish as a second language (L2). According to the Position of Antecedent Strategy (PAS), in native Spanish null pronominal subjects are biased toward subject antecedents, whereas overt pronominal subjects show a “flexible” bias (typically toward non-subject but also toward subject antecedents). The PAS has been extensively investigated in experimental studies, though little is known about real production. We show how naturalistic production (corpus methods) can uncover crucial factors in the PAS that have not been explored in the experimental literature. We analyzed written samples from the CEDEL2 corpus: L1 English-L2 Spanish adult late-bilingual learners (intermediate, lower-advanced and upper-advanced proficiency levels) and a control group of adult Spanish monolinguals (*N* = 75 texts). Anaphors were manually annotated via a fine-grained, linguistically-motivated tagset in UAM Corpus Tool. Against traditional assumptions, our results reveal that (i) the PAS is not a privileged mechanism for resolving anaphora; (ii) it is more complex than assumed (in terms of the division of labor of anaphoric forms, their antecedents and the syntactic configuration in which they appear); (iii) the much-debated “flexible” bias of overt pronouns is apparent since they are hardly produced and are replaced by repeated NPs, which show a clear non-subject antecedent bias; (iv) at the syntax-discourse interface, the PAS is constrained by information structure in more complex ways than assumed: null pronouns mark topic continuity, whereas overtly realized referential expressions (overt REs: overt pronouns and NPs) mark topic shift. Learners show more difficulties with topic continuity (where they redundantly use overt pronouns) than with topic shift (where they normally disambiguate by using overtly realized REs), thus being more redundant than ambiguous, in line with the Pragmatic Principles Violation Hypothesis (PPVH) (Lozano, 2016). We finally argue that the insights from corpora should be implemented into experiments. The triangulation of corpus and experimental methods in bilingualism ultimately provides a clearer understanding of the phenomenon under investigation.

## 1. Introduction: anaphora resolution and the Position of Antecedent Strategy

Anaphora Resolution (AR) is a frequent and pervasive (though deceptively simple) mechanism found in all natural languages. Its acquisition represents a challenge for different types of bilinguals, including late sequential bilinguals like adult second language (L2) learners (Lozano, 2021a).

Anaphors like pronominal subjects refer to their antecedents in discourse. The ambiguous scenario in English (1) requires the resolution of the anaphor: *she* can refer to either antecedent (subject *Carmen* or object *Paola*). Null-subject languages like Spanish are anaphorically more complex since both null (Ø) and overt (*ella* “she”) pronouns can alternate in subject syntactic position, (2), and can refer to either antecedent. Despite this apparent ambiguity, our mental syntactic parser/processor has certain strategies to automatically resolve the anaphor.

(1) Carmen_i_ greeted Paola_j_ while **she**_**i/j**_ was opening the door.
(2) Carmen_i_ saludó a Paola_j_ mientras $\left\{\begin{array}{c} {Ø}_{i/j}\;\\ ella_{i/j}\; \end{array}\right\}$ abría la puerta.

The Position of Antecedent Strategy (PAS),^1^ originally formulated by Carminati (2002) for native Italian, resolves such ambiguity in intrasentential AR (subordinate-main clausal order). Carminati’s results from an offline sentence-interpretation task confirmed this trend: When asked about the interpretation of the second clause (e.g., *Who was in the United States?*), Italian monolinguals chose a subject antecedent (*Marta* 80.72%) with null pronouns in (3), but a non-subject antecedent (*Piera* 83.33%) with overt pronouns. Results from an online self-paced reading task (SPRT) confirmed this: null pronominals (*Ø*) take significantly shorter when referring to preverbal subjects (1,844 ms) than to postverbal objects (2,352 ms), whereas overt pronouns (*lei* “she”) take less time to non-subject (2,236 ms) than to subject (2,266 ms) antecedents.

(3) Marta scriveva frequentemente a Piera quando $\left\{\begin{array}{c} Ø\\ le_{i} \end{array}\right\}$ era negli Stati Uniti.

“Marta wrote frequently to Piera when Ø/she was in the United States.”

The PAS is a syntactic/configurational parsing strategy with a clear division of labor: null pronouns are biased toward a preverbal subject antecedent whereas overt pronouns are biased toward a postverbal non-subject antecedent. Importantly, the PAS is also a syntax-discourse interface phenomenon due to the information status of the anaphor: null pronouns encode a continuation of the preceding subject (topic continuity), whereas overt pronouns mark a topic shift. This holds true in other null-subject languages like Spanish (Lozano, 2009, 2016, 2021a; Martín-Villena and Lozano, 2020), Moroccan Arabic (Bel and García-Alcaraz, 2015), Greek (Prentza and Tsimpli, 2013; Papadopoulou et al., 2015), Croatian (Kraš, 2008a,b), and Romanian (Geber, 2006), among other languages.

The PAS had been extensively investigated in diverse bilingual populations (adult and child L2 learners, heritage speakers, attriters) in different L1-L2 combinations, which has led to the proposal of key theories like Sorace’s (2011) Interface Hypothesis (IH), which predicts bilinguals to show limitations when simultaneously integrating syntactic and discursive information. Follow-up proposals, like Lozano’s (2016) Pragmatic Principles Violation Hypothesis (PPVH), locate the source limitations at a more pragmatic level (topic continuity vs. shift), as a result of the violation of pragmatic principles like Economy vs. Clarity.

Crucially, much of our understanding of AR in general and PAS in particular comes from experimental studies that (i) often report contradictory results, so it is still unclear how the PAS operates in native (and L2) Spanish, and (ii) repeatedly investigate similar anaphoric configuration (i.e., PAS). We argue that highly-contextualized, discourse-rich corpus production data can uncover many factors that have gone undetected in prior experimental studies and solves some of the unresolved PAS questions. Additionally, our developmental corpus data will also allow us to know how the PAS is acquired across proficiency in L1 English-L2 Spanish and whether very advanced learners can eventually acquire the pragmatic subtleties of PAS.

Carminati’s PAS was originally formulated for language processing (comprehension) and our aim is to put it to the test in language production (corpus data). In the psycholinguistic literature, it has long been acknowledged that “grammatical processing (or “parsing”) … refers to the construction of structural representations for sentences, phrases and morphologically complex words in real-time language comprehension and production” (Clahsen and Felser, 2006, p. 564) and that “there may be a closer link between comprehension and production, in particular between parsing and syntactic encoding during production.” (Pickering and van Gompel, 2006, p. 487). In this line, Mac Donald (2013) empirically shows that “language production processes can provide insight into how language comprehension works” (p. 1) and concludes that “the availability of extensive language corpora in many languages permits comprehension researchers to examine the relationship between production patterns (in the corpus) and comprehension behavior” (p. 13). Additionally, it is widely acknowledged in the (bilingual) psycholinguistic literature (e.g., Fernández and Smith Cairns, 2011) that, during processing (parsing), two major processes take place: (i) structuring the incoming input into categories, and (ii) establishing appropriate dependency relations between such categories, which is particularly relevant when there is potential ambiguity (as is the case in PAS scenarios). AR in general and the PAS in particular are classic examples of dependency. Dependencies need to be established not only in comprehension (listener/reader’s perspective), but also in production since the speaker/writer needs to make sure that the anaphoric dependency s/he is producing is configurationally well established and structured (as is the case of PAS scenarios) to ensure that the listener/reader can interpret such dependency and therefore resolve the anaphor. Therefore, the use of production methods (corpora) can shed light on the PAS, as we do in this paper.

We next review the acquisition and processing of PAS in native and L2 Spanish based on experimental and corpus studies (section 1.1). In section 1.2 we present the research questions. The corpus methodology is discussed in section 2. Section 3 presents the results for each research question followed by a discussion, and section 4 concludes with a general discussion/conclusion and future avenues of investigation.

### 1.1. The PAS in native and L2 Spanish

Overall, previous experimental native Spanish PAS findings show no clear division of labor as in native Italian: null pronouns select subject antecedents, but overt pronouns are “flexible” (non-subject and subject antecedents). Each experimental study is unique in terms of, e.g., the type of method/stimuli/design, which could explain the different results across studies. Consequently, we present a thorough review of each study to detect possible limitations that will be later implemented in our corpus study. Note that we review both offline and online PAS studies in adult Spanish monolinguals and adult L2 learners, thereby excluding other populations (see Tables 1, 2 in the online Supplementary material for additional details).^2^ Finally, no single corpus study has targeted PAS structures, so we review some corpus evidence on AR in general as their findings may shed light on PAS.

#### 1.1.1. Offline experimental evidence

Alonso-Ovalle et al. (2002) administered a sentence interpretation task with intersentential PAS (4) to adult Peninsular Spanish monolinguals. Results from the comprehension question (Who is angry?) show a clear subject bias (*Juan* 73.2%) for null pronouns but a “flexible” behavior for overt pronouns (50.2% subject antecedent *Juan*, 49.8% non-subject antecedent *Pedro*), contra Carminati’s (2002) original PAS formulation.

(4)Juan pegó a Pedro. $\left\{\begin{array}{c} Ø\\ él \end{array}\right\}$ está enfadado.

“Juan hit Pedro. (He) is angry.”

Adult Peninsular Spanish monolinguals (with knowledge of Catalan) were tested in an acceptability judgment continuation task, where the plausibility of the continuation sentence (*in italics*) was judged on a four-point scale (Bel et al., 2016a). Monolinguals judged main-subordinate clause order (5) vs. subordinate-main clause order (e.g., *Mientras Javier abandonaba a Pedro, se emborrachó. Pedro se emborrachó*).

(5) Javier abandonó a Pedro miembras se emborrachaba. *Pedro se emborrachaba.*

“Javier abandoned Pedro while (he) was getting drunk. Pedro was getting drunk.”

When both clausal orders are analyzed together, null pronouns refer more to the subject (mean: 3.1) than the object (2.6), but overt pronouns refer to the object (3.2) more than to the subject (2.3). The same holds for *subordinate-main* order (null: 3.55 subject, 2.25 object; overt: 3.25 object, 2.45 subject). This confirms Carminatti’s PAS. In *main-subordinate* order, results for the null pronoun were unexpected (null: 2.71 subject, 3.03 object; overt: object 3.01, subject 2.18). These unexpected monolingual finding led us to incorporate clausal order as a variable in our corpus-based study. The results for monolinguals were similar in Bel and García-Alcaraz (2015), who also included intermediate adult L1 Arabic-L2 Spanish learners in Morocco, both Moroccan Arabic and Spanish being null-subject languages with similar PAS behavior. Learners observed the PAS timidly in both clausal orders: (i) in *main-subordinate*, the null pronouns selected subjects (2.74 in main-subordinate, 2.64 in subordinate-main) slightly more than objects (2.54 and 2.34 respectively), but overt pronouns chose objects (2.81 and 2.63) more than subjects (2.16 and 2.40). In short, learners obey the PAS timidly, whereas Spanish(/Catalan) monolinguals do as well except for the main-subordinate condition, where null pronouns show the opposite behavior.

Jegerski and colleagues conducted a couple of PAS studies. First (Jegerski et al., 2011), they tested L1 English-L2 Spanish adult learners (intermediate, advanced) and adult Spanish monolinguals (from Spain and Latin America) in an ambiguous PAS sentence-interpretation task with null and overt pronouns (6).^3^

(6)Marta le escribía frecuentemente a Lorena cuando $\left\{\begin{array}{c} Ø\\ \mathrm{ella} \end{array}\right\}$ estaba en los Estados Unidos.

“Marta wrote frequently to Lorena when (she) was in the United States”.

When asked about the anaphoric interpretation, monolinguals preferred to link null pronouns with subject antecedents (75%), as predicted by the PAS, but overt pronouns show again a “flexible” behavior (53% subject antecedents, 47% object antecedents). Advanced learners show a native-like tendency: null-subject 69%, and “flexible” overt pronoun behavior (56% subject antecedent, 44% object antecedent). Intermediates show a timid subject bias irrespective of the pronoun type (null-subject 66%, overt-subject 60%). In their second study, Keating et al. (2011) employed the same methodology and the same profiles of participants. Once again, Spanish monolinguals significantly preferred a null pronoun (74%) to an overt pronoun (54%) to refer to the subject. By contrast, the difference was not significant in advanced learners (60.15% null vs. 54.21% overt). Results from both studies indicate that overt pronouns show a “flexible” behavior by referring around 50% of the time to the subject and 50% to the object, both in native and L2 Spanish, a fact to which we will return in our study.

In a picture-verification task, Clements and Domínguez (2017) tested the PAS in adult monolinguals (mainly from Spain, some from Mexico) and advanced L1 English-L2 Spanish learners from the United Kingdom, who were presented with two pictures and a PAS sentence with(out) an overt pronoun, as in (6). They had to decide whether the given sentence corresponded to one or the other picture (or both). Monolinguals preferred to link a null pronoun with a subject (77%) more than an object (12%) antecedent, whereas overt pronouns showed the opposite pattern (54% object, 27% subject), which supports Carminati’s original PAS formulation, though note once again that the intuitions for overt pronouns are not as strong as those for null pronouns, a fact to which we will return in this paper. Unlike previous findings above, advanced learners observed the PAS in a native-like manner (null: subject 68%, object 21%; overt: object 63%, subject 23%).

Chamorro et al. (2016) asked adult monolinguals from Spain to rate null/overt pronoun PAS under four conditions: two forced antecedent-subject biases (singular subject, plural object (7a)), and two forced object-antecedent biases (plural subject, singular object (7b)). Monolinguals non-significantly rated the null pronoun to equally refer to the subject (3.72) and the object (3.61) antecedent, showing no clear subject bias of null pronouns, which runs against all the findings reviewed above. The overt pronoun significantly referred to the object (3.60) more than the subject (3.26) antecedent (though note the 3.26 vs. 3.60 ratings are not different enough given the 1–5 Likert rating scale).

(7) a. La madre_i_ saludó a las chicas_j_ cuando $\left\{\begin{array}{c} {Ø}_{i}\\ {\mathrm{ella}}_{i} \end{array}\right\}$ cruzaba una calle con mucho tráfico.
b. Las madres_i_ saludaron a la chica_j_ cuando $\left\{\begin{array}{c} {Ø}_{j}\\ {\mathrm{ella}}_{j} \end{array}\right\}$ cruzaba una calle con mucho tráfico.

“The mother (s) greeted the girl (s) when (she) was crossing a street with lots of traffic”.

In a picture selection task, Martín-Villena (2023) tested conjunction type (when vs. while) in Peninsular Spanish monolinguals in sentences like (6). Subject-antecedent preferences with conjunction *cuando* “when” were higher for null (67%) than overt (23%) pronouns as well as with *mientras* “while” (null: 80%; overt: 30%). This confirms PAS preferences for subject antecedents but shows that null-subject bias was somewhat stronger with mientras “while” than with cuando “when”.

#### 1.1.2. Online experimental evidence

All online experiments to date have used SPRT, which measure reading times (RTs) in milliseconds (ms). Filiaci (2010) was the first online study to test PAS in Peninsular Spanish monolinguals. In intrasentential subordinate-main clauses, (8), the semantics of the main clause forced the anaphor toward the subject (8a) or the object (8b) antecedent. RTs of the main clause with a null pronoun were significantly faster when biasing toward the subject (1,998 ms) than the object (2,319 ms) antecedent, as predicted by Carminati’s PAS, but with an overt pronoun, RTs were faster when biasing toward the object (2,389 ms) than the subject (2,507 ms) (but differences were non-significant, which reflects again the “flexible” behavior of Spanish overt pronouns). These results were later published (Filiaci et al., 2014) as experiment 1. Experiment 2 stimuli were the same as in experiment 1 but RT analyses were conducted at different phrasal regions (separated by slashes “/” in (9)). Overall, results replicated those found in experiment 1, thus confirming the “flexibility” of overt pronouns in Spanish when compared to Italian.

(8) a. Cuando Ana_i_ visitó a María_j_ en en el hospital, $\left\{\begin{array}{c} {Ø}_{i}\\ {\mathrm{ella}}_{i} \end{array}\right\}$ le llevó un ramo de rosas.
b. Cuando Ana_i_ visitó a María_j_ en en el hospital, $\left\{\begin{array}{c} {Ø}_{j}\\ {\mathrm{ella}}_{j} \end{array}\right\}$ ya estaba fuera de peligro.

“When Ana visited Mary in the hospital, (she) {brought her a bunch of roses | was already out of danger.}”

(9) Cuando / Ana / visitó / a María / en en el hospital, / $\left\{\begin{array}{c} {Ø}_{i}\\ {\mathrm{ella}}_{i} \end{array}\right\}$ / le llevó / un ramo / de rosas.

Gelormini-Lezama and Almor (2011) tested intersentential PAS with adult Argentinian Spanish monolinguals. Sentences also included repeated names (RNs) (e.g., *Juan* “John”), (10). The object clitic (*la* “her”) forces the null pronoun toward a subject (10a) or object (10b) antecedent reading. With forced subject antecedents, RTs for null-pronoun sentences (1,812 ms) were faster than overt-pronoun sentences (2264), but the opposite was true when with forced object antecedents (null 2,412, overt 2,157). This clearly confirms Carminati’s PAS prediction. Interestingly, RNs were read equally fast irrespective of their antecedent (2080 subject, 2055 object) and their RTs did not significantly differ from sentences containing overt pronouns but did significantly differ from sentences containing null pronouns (subject: null < RN; object: null > RN), which suggests that NPs may play a role in object-antecedent selection in AR in native Spanish, a fact to which we will return in our corpus analysis.

(10) a. Juan_i_ se encontró con María_j_. $\left\{\begin{array}{c} {Ø}_{i}\\ Él_{i}\\ {\mathrm{Juan}}_{i} \end{array}\right\}$ la_j_ vio triste.
b. María_i_ se encontró con Juan_j_. $\left\{\begin{array}{c} {Ø}_{j}\\ Él_{j}\\ {\mathrm{Juan}}_{j} \end{array}\right\}$ la_i_ vio triste.

“{John found Mary | Mary found John}. Ø/He/John found her sad.”

Another study (Bel et al., 2016b) tested adult Peninsular Spanish monolinguals in intrasentential (main-subordinate order) PAS, (11), presented in a word-by-word, non-cumulative fashion. The ambiguous anaphor is resolved postverbally via world knowledge: *violin* forces a subject antecedent (musician), whereas *casco* “helmet” forces an object antecedent (firefighter).

(11) El músico_i_ saluda al bombero_j_ mientras $\left\{\begin{array}{c} {Ø}_{i}\\ él_{i} \end{array}\right\}$ lleva $\left\{\begin{array}{c} \mathrm{un}\;\mathrm{viol}ín\\ \mathrm{un}\;\mathrm{casco} \end{array}\right\}$ en la mochila.

“The musician greets the fireman while (he) carries {a violin | a helmet} in his backpack.”

Null pronouns were read significantly faster with a subject-antecedent bias (798 ms) than an object-antecedent bias (887 ms) at the NP object region (*un violin/un casco*), but not at the locative PP region (*en la mochila*) (1,143 vs. 1,453 ms). By contrast, overt pronouns were read significantly faster with an object-antecedent bias (1,308 ms) than with a subject-antecedent bias (1,402 ms) at the PP region, but not at the object region (884 vs. 887 ms). Findings are in line with Carminatti’s PAS prediction, though note that (i) RT differences^4^ for overt pronouns (170 ms) are smaller than for null pronouns (399 ms), which suggests again a rather “flexible” antecedent bias for overt pronouns; (ii) RT differences are more observable in some regions than in others, which suggests that these stimuli are not straightforwardly parsed probably due to the complex disambiguation mechanism. Further results from adult L1 Arabic-L2 Spanish and L1 English-L2 Spanish learners at three proficiency levels (intermediate, upper intermediate, high) revealed that the advanced learners can eventually parse PAS structures in a native-like fashion, irrespective of their L1 (a (non)null-subject language like English or Arabic).

Intrasentential (subordinate-main order) PAS was investigated in adult Mexican Spanish monolinguals (clause-by-clause presentation) (Keating et al., 2016). The ambiguous anaphor is resolved postverbally via world knowledge: *su culpabilidad* “his guilt” forces a subject antecedent (*el sospechoso* “the suspect”) in (12a), but an object antecedent in (12b). Null-pronoun clauses were read significantly faster with subject (2,186 ms) than with object (2,447 ms) antecedents. By contrast, overt-pronoun sentences were read faster with object (2,456 ms) than with subject (2,605 ms) antecedents. These results confirm Carminatti’s PAS but note that if we calculate the RT differences,^5^ the mathematical difference is smaller for overt pronouns (194 ms) than for null pronouns (261), which suggests again a certain “flexibility” for overt pronouns.

(12) a. Después de que el sospechoso_i_ habló con el policía_j_, $\left\{\begin{array}{c} {Ø}_{i}\\ él_{i} \end{array}\right\}$ admitió su culpabilidad.
b. Después de que el policía_i_ habló con el sospechoso_j_, $\left\{\begin{array}{c} {Ø}_{j}\\ él_{j} \end{array}\right\}$ admitió su culpabilidad.

“{After the suspect talked to the policeman | After the policeman talked to the suspect}, (he) admitted his guilt.”

In a SPRT, Martín-Villena (2023) used the same stimuli as in the offline experiment above. Results showed differences depending on the region analyzed. In the subordinate clause segment, null pronouns showed an unclear bias (Subject: 1,383 ms; Object: 1,372 ms), but overt pronouns exhibited a clear object bias (Subject: 2,129 ms; Object 1,940 ms). Interestingly, in the comprehension question segment, null pronouns showed a subject bias (Subject: 946 ms; Object 1,051 ms), but overt pronouns showed an object bias (Subject 1,242 ms; Object: 1,037 ms), as predicted by the PAS.

#### 1.1.3. Summary of the experimental evidence: native Spanish

The native Spanish PAS results from the experimental studies are often contradictory. This could be due to multiple factors (many of which were taken into account in our corpus study), e.g.: the different varieties of the monolinguals of Spanish; the PAS configuration (intersentential vs. intrasentential) and the clausal order (main-subordinate vs. subordinate-main); and the different formats (and presentation types) of the offline and online experimental methods, among others.

**Figure 1:**
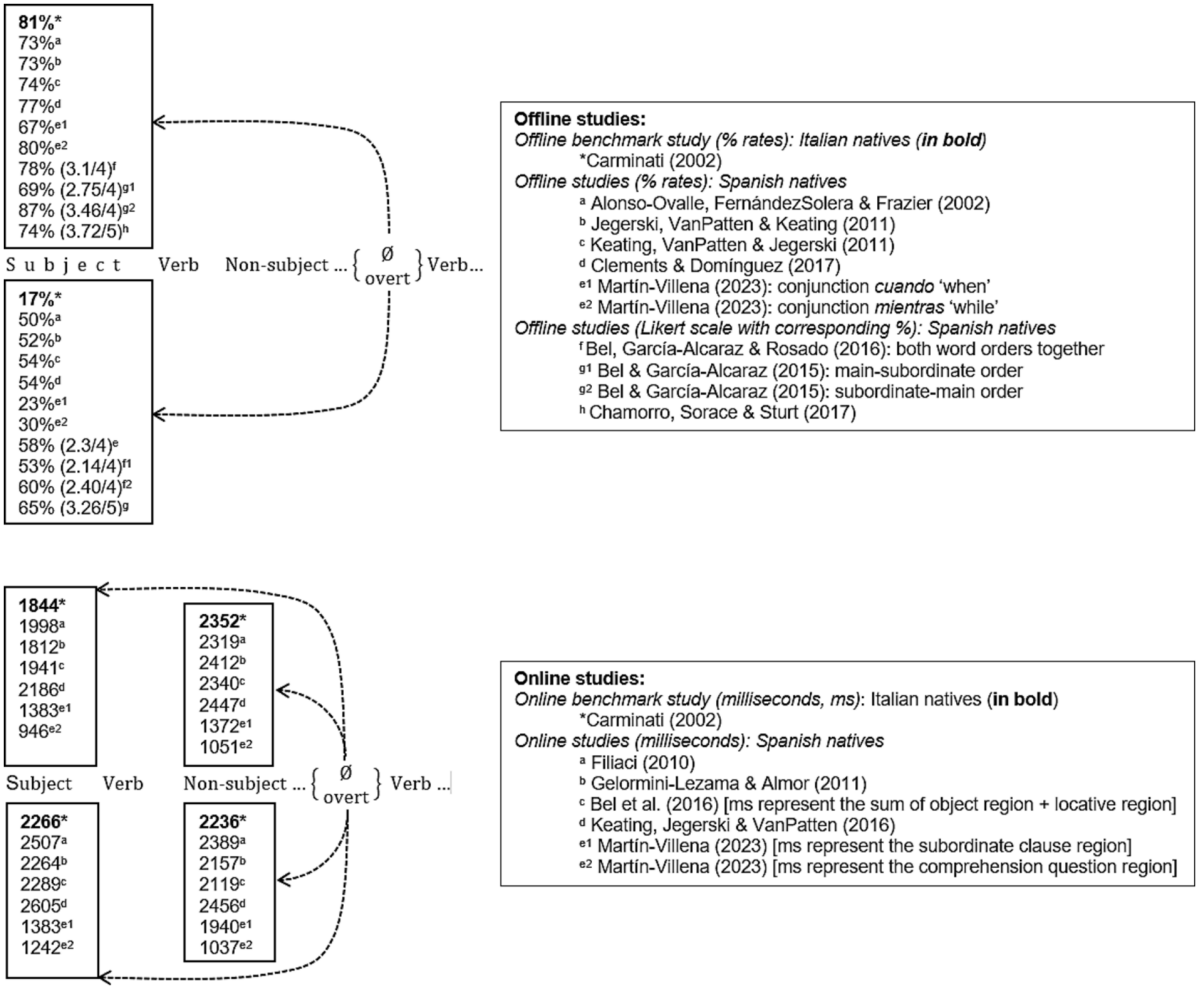
Summary of offline and online preferences for the PAS in native Spanish and Italian.

A visual summary of *offline* PAS biases in native Spanish (Figure 1) suggests that the original PAS formulation for native Italian is not fully operative in native Spanish: Whereas null pronouns clearly select a subject antecedent (69% ~ 87% range), as predicted by Carminati’s PAS, overt pronouns show a “flexible” preference by often selecting an object antecedent around half of the time (50% ~ 65% range), which implies that the rest of the time they select a subject antecedent. In *online* experiments, null-subject sentences are read significantly faster with forced subject than with forced object antecedents, whereas overt-subject sentences are read faster with forced object than subject antecedents, as predicted by PAS, though note that the subject vs. object RT differences are usually weaker with overt pronouns than with null pronouns, which again suggests a mild “flexibility” of overt pronouns. The offline and online native Spanish findings thus suggest that, whereas null pronouns have a strong subject bias, overt pronouns are less clear-cut (i.e., more “flexible”) in their choice of antecedent. We will argue that such flexibility is more apparent than real, as our corpus data will reveal.

#### 1.1.4. Corpus evidence

To our knowledge, there is no corpus-based study targeting specifically the PAS in adult Spanish monolinguals/learners. At best, there is some indirect PAS evidence since the corpus studies to be reviewed analyzed multiple types of AR scenarios (including PAS), so it is unclear to what extent their findings can extrapolate to specific PAS scenarios.

The experimental study reviewed above (Bel et al., 2016a) presents additional evidence from a written and spoken production task by Peninsular Spanish monolinguals. The researchers analyzed different types of AR scenarios, including PAS-like scenarios. Null pronouns clearly biased toward subject antecedents (77.27%), while overt pronouns showed a less clear-cut antecedent bias (subject: 42.86%; non-subject: 57.14%). Moroccan Arabic/Spanish early bilinguals’ null pronouns clearly biased toward subject antecedents (70.19%), while their overt pronouns biased toward both non-subject (35.71%) and subject (64.29%) antecedents. Overt pronouns reflect again the already reported “flexibility”. Importantly, this study (i) does not report the production of NP anaphors, which are crucial for our understanding of AR in general and the PAS in particular, as we will later show in this paper; (ii) analyses both singular and plural anaphoric forms together, though corpus data has shown that only 3rd singular anaphors are problematic for learners (Lozano, 2009, 2016); and (iii) presents data from teenage Spanish monolinguals ^6^ and early bilinguals, so the evidence about how the PAS operates in adult monolinguals and L2 Spanish is rather indirect. In a follow-up study, García-Alcaraz and Bel (2019) used the same task and coding criteria. This time, the Spanish monolinguals were university students, and the L1 Moroccan Arabic-L2 Spanish learners were teenage sequential bilinguals. Results suggest that both monolinguals and bilinguals produce null pronominal subjects to mark topic continuity around 2/3 of the time and topic shift around 1/3. Regarding overt pronouns, their production was very low (4 tokens or less depending on the configuration), which is not very informative. In short, while suggestive, these findings do not fully inform about PAS scenarios in either native or L2 adult Spanish.

A series of corpus studies (Lozano, 2009, 2016; Martín-Villena and Lozano, 2020) targeted AR scenarios with subject anaphoric forms (null/overt pronouns, as well as NPs). Results from adult Peninsular Spanish monolinguals reveal some consistent findings across studies: whereas null pronouns clearly encode topic continuity, it is NPs that encode topic shift more often than overt pronouns do, particularly when there are several potential antecedents in discourse. L1 English-L2 Spanish learners do not typically show problems in topic-shift contexts (as they use overt forms to avoid ambiguity) but are redundant in topic-continuity contexts (as they overuse overt pronouns). These findings are captured by the Pragmatic Principles Violation Hypothesis (PPVH) (Lozano, 2016), which postulates differential effects at the syntax-discourse interface with AR: learners obey the pragmatic Principle of Clarity as they use full anaphoric forms in cases of ambiguity, but they are lax with the Principle of Economy, as they redundantly produce overt anaphoric forms when not required in topic continuity, though can be modulated by the amount of potential antecedents. In short, learners are more redundant than ambiguous. We will get back to the PPVH when discussing our results.

To summarize, the corpus-based findings are clearly insufficient since they: (i) do not specifically target PAS scenarios but rather conflate different types of AR scenarios in their analyses; (ii) some of them do not consider the role of subject NPs as an anaphoric form in its own right. This, coupled by certain limitations in the experimental studies, motivated the formulation of our research questions with a view to answering some unresolved issues in the production of PAS in native and L2 Spanish.

### 1.2. The current study: research questions and hypotheses

The bulk of experimental studies on AR have investigated the PAS with two potential antecedents (subject/non-subject) and two anaphoric forms (overt/null pronominal subject) in either inter- or intra-sentential configurations. So, what we know about the PAS comes mostly from a series of similarly-designed experiments that do not question whether (i) the PAS may represent an oversimplified way of resolving anaphora in native (and L2) Spanish; (ii) PAS scenarios may be more complex than traditionally assumed (i.e., they can contain more than two antecedents in other syntactic positions); (iii) the antecedents may be realized by other forms other than null/overt pronouns (i.e., NPs for example). Unlike experiments, corpus data can shed light on these questions since they contain natural language production (where AR configurations are neither controlled nor constrained) and offer contextually rich scenarios with anaphors and antecedents embedded in their entire discourse. Unlike experiments, corpus data can shed light on these questions since they (i) contain natural language production where AR configurations are neither controlled nor constrained; (ii) offer contextually rich scenarios with anaphors and antecedents embedded in their entire discourse. This led to *RQ1a* and *RQ1b*.

*RQ1a* (*Prototypicality of PAS*): Is the PAS a prototypical way of resolving anaphora in native (and in non-native) Spanish, as implicitly assumed in the literature?

*H1a*: The PAS is but one of many possible mechanisms for resolving anaphors in native and non-native Spanish.

*RQ1b* (*Complexity of PAS*): Can the standard PAS configuration (subject/non-subject antecedent; null/overt pronominal subject anaphor) be more complex than assumed in the literature?

*H1b*: Corpus data will reveal that the PAS is richer than standardly assumed, in terms of antecedent configurations, syntactic possibilities and range of anaphoric forms.

Experimental PAS studies have typically restricted their focus to two anaphoric forms (overt/null pronominal subjects). Corpus studies have reported the use of other anaphoric forms (e.g., repeated Ns and NPs) in several AR scenarios, so NPs may be also possible Refererential Expression (RE) forms in PAS.^7^

*RQ2* (*RE forms in discourse*): Apart from null/overt pronominal subjects, are other RE forms possible in native and L2 Spanish PAS?

*H2*: In line with corpus findings on AR in general, we predict for PAS (i) null pronouns to be abundant due to the null-subject nature of Spanish; (ii) overt pronouns to be infrequent and, (ii) importantly, NPs to be more frequent than overt pronouns. The range of REs in PAS will therefore include null/overt pronominal anaphors and NPs (used with an anaphoric value).

Experimental studies report Spanish null pronouns to bias toward a preverbal subject antecedent, whereas overt pronouns show a more “flexible” behavior. This contrasts with native Italian where overt/null pronouns show a clear division of labor. Additionally, experimental studies have not typically included NPs as a possible RE form.

*RQ3* (*Division of labor*): Regarding the division of labor in native and L2 Spanish, will the “flexible” behavior of overt pronouns be better accounted for if NPs are also included as a possible type of RE?

*H3*: Null pronouns will be clearly biased toward a subject antecedent, as previously reported, whereas overtly realized REs (i.e., overt pronouns and NPs together) will be clearly biased toward non-subject antecedents. Learners will show growing sensitivity to such division of labor as proficiency increases, but native-like ultimate attainment is not expected for upper-advanced learners since the PAS is constrained at the syntax-discourse interface (cf. *RQ4* below), which is a problematic area for L2 learners (Lozano, 2021a for an overview).

The implicit assumption in the experimental literature is that purely configurational factors (null➔subject vs. overt➔non-subject) overlap with discursive information-status factors (null➔topic continuity vs. overt➔topic shift). *RQ4*/*H4* (when contrasted to *RQ3*/*H3*) will determine the extent to which the overlap assumption is correct. This motivates theoretical questions having to do with likely deficits at the syntax-discourse interface.

*RQ4* (*Syntax-discourse interface*): Will syntactic configuration overlap with information status in PAS configurations and, if so, will learners be eventually (un) able to acquire this syntax-discourse phenomenon?

*H4*: Syntactic configuration will overlap with information status and NPs will play a role (null➔subject/topic continuity; overt & NP➔non-subject/topic shift). Learners will show an increasing trend toward the native norm, yet the syntax-discourse properties of the PAS will not be fully acquired, as predicted by models like the IH and the PPVH.

Despite English being a non-null subject language, corpus data (Quesada and Lozano, 2020) have shown that English monolinguals allow null pronouns in very specific contexts: topic continuity and coordination at around 77% (e.g., *Lucy_i_ walked for an hour and Ø_i_ had a picnic*), but never in non-coordinate configurations. So, it could be argued that L2 Spanish learners’ production of null pronouns in topic continuity could be due to L1 transfer rather than actual acquisition, which leads to the following exploratory research question.

*RQ5* (*Cross-linguistic influence*): Will L2 Spanish learners’ distribution of null pronouns be a reflection of their allowance in their L1 English (topic continuity and coordination) or will it be a reflection of acquisition at the syntax-discourse interface? It may be the case that learners transfer in initial stages but progressively acquire the discursive distribution of null pronouns.

*H5*: (*Transfer account)*

If L2 Spanish learners are transferring from their L1 English, null subjects will be produced mainly where they are allowed in English (topic continuity with coordination) and not where they are not allowed (topic continuity with non-coordination).

(*Non-transfer account*, i.e., acquisition account)

If they are rather sensitive to the pragmatics of null pronouns in Spanish, null subjects will be produced where they are allowed in native Spanish, i.e., across the board (both in coordination and non-coordination).

Previous PAS experimental studies are often contradictory depending on the sentential configuration: inter- vs. intra-sentential; main-subordinate vs. subordinate-main orders (*cf.* the tables in the online Supplementary material). *RQ6* is an exploratory question to explore whether the sentential PAS configuration modulates the choice of RE in naturalistic corpus production.

*RQ6* (*Sentential configurations*): In which sentential configurations (intra- vs. inter-sentential) will PAS structures be more frequent in naturalistic corpus production? Which PAS clausal order (main-subordinate vs. subordinate-main) is prototypical? Will learners’ production ultimately approach to/deviate from Spanish monolinguals?

## 2. Method

### 2.1. Corpus: CEDEL2

*Corpus Escrito del Español L2* (CEDEL2) (Lozano, 2022) is a multi-L1 corpus of L2 Spanish learners coming from 11 different L1 backgrounds, plus a Spanish monolingual control subcorpus. CEDEL2 (version 2) currently holds 1,105,936 words, 4,399 participants, and 14 task topics. It is freely available/downloadable at http://cedel2.learnercorpora.com.

Data are collected via online forms^8^ and participants complete three forms: (i) linguistic background; (ii) standardized placement test (just for learners) (University of Wisconsin, 1998); and (iii) written/spoken text.

### 2.2. Sample

We selected an L1 English-L2 Spanish (plus a comparable Spanish monolingual control) sample (Table 1) based on the following criteria: (i) the participant’s age range was 18 ~ 40, since Working Memory, which may affect AR, appears to decay after the age of 40 (Bel et al., 2016b); (ii) learners’ proficiency-level range was intermediate~advanced; and (iii) only two composition titles were targeted (*cf.* 2.3 below).^9^ Two hundred two texts met these criteria but we finally selected those that had at least one instance of a PAS (*N* = 75). We originally departed from two intermediate groups: lower intermediates (placement score: 21 ~ 28 raw score, 49% ~ 65%) and upper intermediates (29 ~ 35, 67–81%). Since they did not significantly differ in our analyses, we decided to analyze both groups as a single group of intermediates to simplify the between-group statistical analyses and interpretations. Learners had an equivalent age of exposure (AoE) to L2 Spanish and their length of instruction (LoI) in Spanish and length of stay abroad (LoSA) in a Spanish-speaking country increased with proficiency.

**Table 1:** Texts and participants’ bio-data.

Group	Intermediate		Lower advanced	Upper advanced	Monolinguals
	Lower	Upper			
Placement raw score (0–43)	21 ~ 28	29 ~ 35	36 ~ 40	41 ~ 43	–
Equivalent percentage (0–100%)	49 ~ 65%	66 ~ 81%	82 ~ 94%	95 ~ 100%	–
Texts that met criteria	69		37	13	103
Texts analyzed	21		19	8	27
Mean age	20.8		21.3	25.5	25.6
Mean proficiency	69.6%		86.8%	96.7%	–
AoE (years)	14		14.5	12.2	–
LoI (years)	5.3		5.5	8.8	–
LoSA (months)	7.4		11.1	12.3	–

### 2.3. Task

We selected two task tittles (*Talk about a famous person* and *Summarize a film you have recently seen*), since they are narratives that contain (i) abundant [+human] 3^rd^ person antecedent-anaphor chains; and (ii) PAS constructions, which were more frequent in the second task than in the first task and which offered different characters in discourse suitable for the topic continuity/shift purpose of this study.

### 2.4. Corpus annotation and tagset

**Figure 2:**
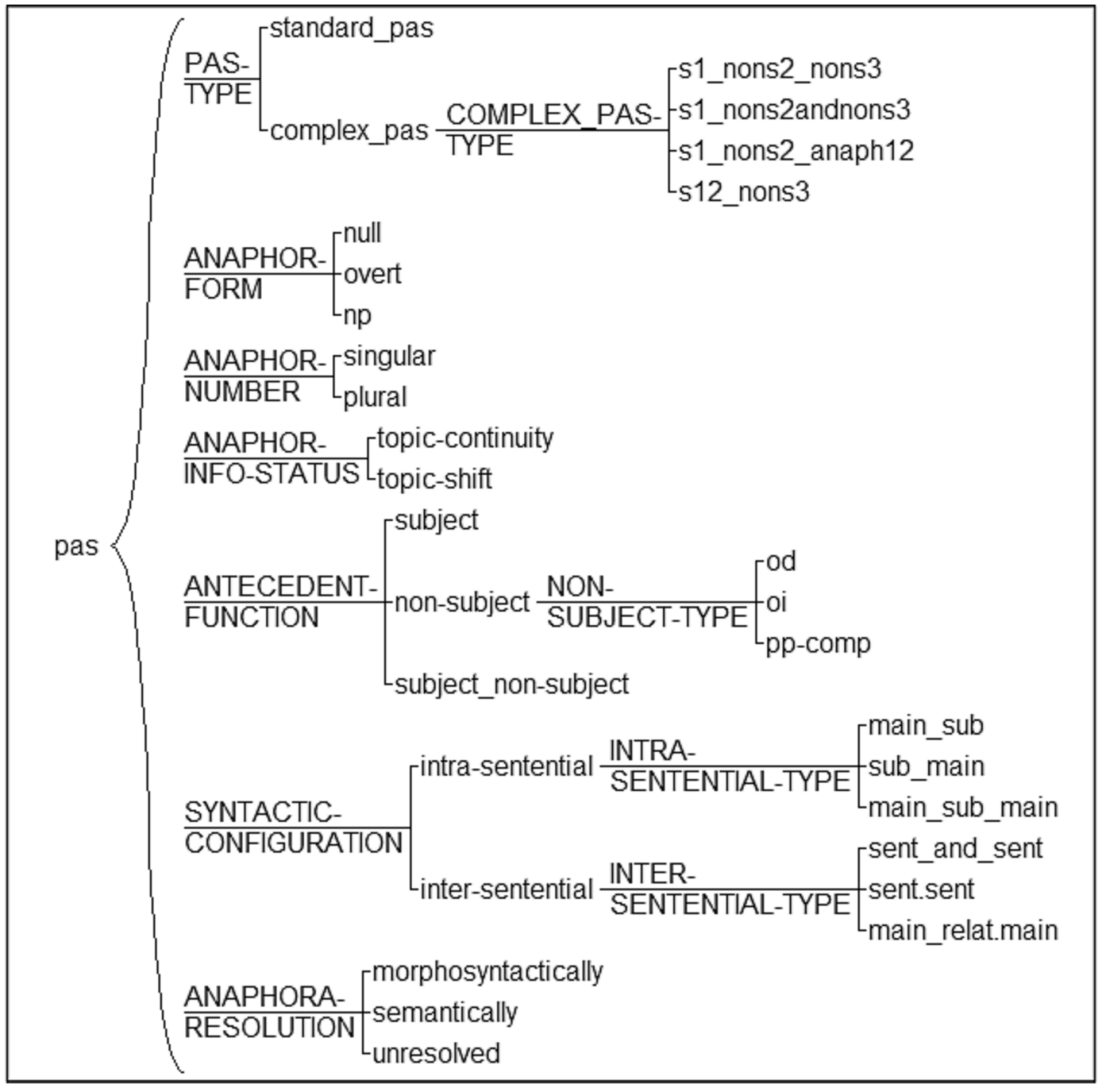
PAS annotation tagset.

The corpus sample was manually annotated (i.e., tagged) with UAM Corpus Tool (O’Donnell, 2009), version 6.2j (February 2023).^10^ We firstly tagged each text to indicate the group category (intermediate, lower advanced, upper advanced, and monolingual), which allows between-group comparisons for the same linguistic feature, as will be explained below. We designed another tagset to count the frequency of two AR scenarios (PAS vs. other AR). Each RE in subject position was assigned either the *PAS* tag (when the RE was preceded by a subject/non-subject antecedents) or *other* (when the RE was preceded by an AR scenario other than PAS). Figure 2 shows the fine-grained, linguistically-informed tagset to annotate PAS.^11^ It allows for multiple and intricate statistical analyses among tags, as will become obvious later. It is inspired by previous corpus studies on AR (Lozano, 2016; Quesada and Lozano, 2020), although we introduced new features.

Every 3rd person human subject that followed the syntactic configuration of the PAS was manually tagged. First, the **PAS-type** system included: (i) *standard* PAS with two antecedents, as in (13), and (ii) *complex* PAS with more than two antecedents, as in (14a-c). For example, the tag used to annotate complex PAS in (14a) is *s1_nons2_nons3*, which indicates we have 3 potential antecedents: the first one is in subject position (s1) and the other two in non-subject position realized via a complex NP: PP (nons3) within an NP (nons2). In (14b), the tag *s1_nons2andnons3* indicates that there is a singular antecedent in subject position (s1, which happens to be a null pronouns) followed by two NP coordinated antecedents in non-subject position (s2&s3) embedded within a PP. Notice that, due to the complexity of the antecedents’ region, the anaphor is a complex NP for disambiguation purposes. Other complex PAS contained plural REs, as in (14c), but we excluded them from our current analysis since it has been shown that the truly problematic cases of AR are 3rd person singular and not plural (Lozano, 2009).

(13) Standard PAS:

*Naaven_i_* se ha enamorado de *Tiana_j_* y **Ø**_**i**_ quiere pedirle matrimonio. [Monolingual: ES_WR_24_3_IZG.txt].^12^

“Naaven_i_ has fallen in love with Tiana_j_ and Ø_i_ wants to propose to her”.

(14) Complex PAS:

a.*La chica_i_ se* enamora del *amante_j_* de su *madre*k** hasta que al final Ø_**i**_ acaba teniendo … [Monolingual: ES_WR_30_3_JVM].

“The girl_i_ falls in love with the lover_j_ of her mother_k_ until Ø_i_ ends up having…”.

- Pero el principal problema que **Ø**_**i**_ tenía era que **Ø**_**i**_ sufría un maltrato constante por parte de su **madre**_**j**_ y del **novio**_**k**_ de **ésta**_**j**_. **El novio**_**k**_ **de la madre**_**j**_ había… [Monolingual: ES_WR_31_3_EAC]

“But the main problem Ø_i_ had was that Ø_i_ was abused by her mother_j_ and the boyfriend_k_ of her_j_. The boyfriend_k_ of the mother_j_ had…)”.

- *Ø_ij_* Juntos tendrán que huir de *Dr. Facilier_k_* a los pantanos, dnde **Ø**_**ij**_ se encuentran… [Monolingual: ES_WR_24_3_IZG]

“Ø_ij_ Together will have to escape from Dr. Facilier_k_ to the swamps, where **Ø**_**ij**_ meet …”.

The **anaphor-form** system includes the RE form (null/overt pronouns and NPs) in subject position, as shown in bold in (15). The **anaphor-number** system includes the RE number (singular/plural), which served us to exclude plural REs in the analyses, as justified above.

(15) … **el protagonista**_i_ de la película se enamora de la chica_j_ y **ella**_j_ le_i_ pide por favor que **Ø**_i_ deje el negocio … [Monolingual: ES_WR_23_3_EM].

“…the **main character**_i_ of the film falls in love with the girl_j_ and **she**_j_ asks him_i_ that **Ø**_i_ leaves the business …”

The **information-status** system comprises topic-continuity and topic-shift contexts, as in (16a-b) respectively. The **antecedent-function** system included subject antecedent, non-subject antecedent, and subject/non-subject antecedent (for cases of complex PAS). This system allowed us to detect PAS scenarios with subject-antecedent biases, as in (16a), or non-subject antecedent biases, as in (16b).

(16) a. Un periodista_i_ investiga la desaparición de una rica heredera_j_, hace cuarenta años. Para ello, **Ø**_i_ cuenta con…
  [Monolingual: ES_WR_24_3_AW].

“A journalist_i_ investigates the disappearance of a rich heiress_i_, 40 years ago. To do so, **Ø**_i_ relies on…”

b. Bella_i_ se da cuenta de que Jacob_j_ está enamorado de ella_i_ y **ella**_i_ también un poco de él_j_ [Monolingual: ES_WR_21_3_ICH].

“Bella_i_ realizes that Jacob_j_ is in love with her_i_ and **she**_i_ is also in love with him_j_”.

In the **syntactic-configuration**, we tagged the type of intra-sentential and inter-sentential configurations, e.g., topic-continuity and coordination in (16b) and topic continuity and non-coordination, which can be of different types, e.g., subordination in (17) or new sentence in (18).

(17) … un padre_i_ trata por todos los medios de llevar a su hijo_j_ de diez años hasta el mar, donde **Ø**_i_ espera encontrar… [Monolingual: ES_WR_22_3_AFL].

“…a father_i_ tries by all means to take his ten-year-old son_j_ to the sea, where **Ø**_i_ hopes to find …”

(18) … y Ø_i_ llega a cortarle_j_ un dedo de un hachazo. Después **Ø**_i_ intenta matar a George_k_… [Monolingual: ES_WR_28_3_MAAO].

“… and Ø_i_ cuts off her_j_ finger with an axe. Later Ø_i_ tries to kill George_k_…”.

Finally, the **anaphora-resolution** system indicates the type of resolution: via morphosyntax or semantics. In this paper, we analyzed only the PAS that was morphosyntactically resolved. In order to avoid skewing our results, we excluded PAS that was semantically resolved (i.e., null pronouns in topic-shift scenarios like (19), which are ultimately resolved via directive verbs).

(19) Ella_i_ le_j_ pide que **Ø**_j_ espere a…[Monolingual: ES_WR_26_3_MPVI].

“She_i_ asks him_j_ that **Ø**_j_ waits for her_i_ to…”.

### 2.5. Analysis

UAM Corpus Tool has an in-built statistical analysis software. Between-group (or between-system/tag) comparisons are based on the tags’ raw frequencies and statistical contrasts are chi square (*χ*^2^) tests, accompanied by their significance level (*p*) and their effect size (Cohen’s *h*).

Based on the linguistically-motivated tagging scheme (Figure 2), UAM Corpus Tool allows for multiple and sophisticated statistical contrasts between the different groups and the (sub) nodes and terminal nodes of the tagset. These contrasts were motivated by the linguistically-informed hypotheses from section 1.2. Following statistical recommendations for corpus data (Egbert et al., 2020), we purposely decided to use the *χ*^2^ statistical contrasts provided by the software rather than submitting the data to more sophisticated statistical analyses (which involve transforming the data and abstracting away from the linguistic facts and interpretations):

“the most appropriate method for the task at hand should not be the most sophisticated method … Instead, we should always strive to choose minimally sufficient statistical methods, meaning that we should choose tests that are no more nor less sophisticated than the study design requires. The reason for this is twofold: (1) all descriptive and inferential statistical tests force us to abstract away from language to some extent and (2) there is often an inverse relationship between the level of sophistication of the method and the linguistic interpretability of the results.” (Egbert et al., 2020, p. 40)

## 3. Results and discussion

We next present and discuss the results for each research question. We leave the general discussion for section 4.

### 3.1. *RQ1*/*H1*: frequency of PAS scenarios in natural language production

**Figure 3:**
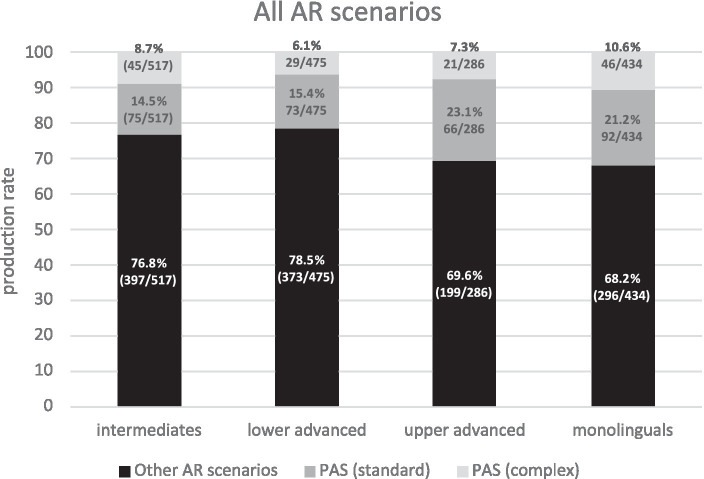
AR scenarios by group.

In Figure 3, PAS scenarios (gray bars) were compared against other types of AR scenarios (black bars). Spanish monolinguals resolve anaphora via scenarios (68.2%, i.e., 296 REs out of a total of 434 tagged REs) other than standard PAS (21.2%) or complex PAS (10.6%). Thus, standard PAS only amounts to around 1/5th of the total possible AR scenarios. Learners show a similar pattern to monolinguals across all proficiency levels, though only the upper advanced group shows native-like behavior (standard and complex PAS *χ*^2^ = 2.16, *p* = 0.1419 n.s., *h* = 0.204; other scenarios *χ*^2^ = 0.15, *p* = 0.6964 n.s, *h* = 0.030). The lower-level learner groups significantly differ from Spanish monolinguals in other scenarios but not in standard and complex PAS scenarios (intermediates vs. monolinguals: standard and complex PAS *χ*^2^ = 0.49, *p* = 0.4848 n.s., *h* = 0.087, other scenarios *χ*^2^ = 8.80 *p* = 0.0030, *h* = 0.193; lower-advanced vs. monolinguals: standard and complex PAS *χ*^2^ = 0.66, *p* = 0.4180 n.s., *h* = 0.106, other scenarios *χ*^2^ = 12.44, *p* = 0.0004, *h* = 0.235).

Our findings support *H1a* (PAS represents one of the many possible mechanisms of AR in native and non-native Spanish) and *H1b* (PAS can contain more complex configurations than those traditionally reported in the literature). Corpus data reveal that the traditional assumption of standard PAS as a prototypical strategy to resolve anaphora has been overestimated in the experimental literature.

### 3.2. *RQ2*/*H2*: overall use of REs in PAS scenarios

**Figure 4:**
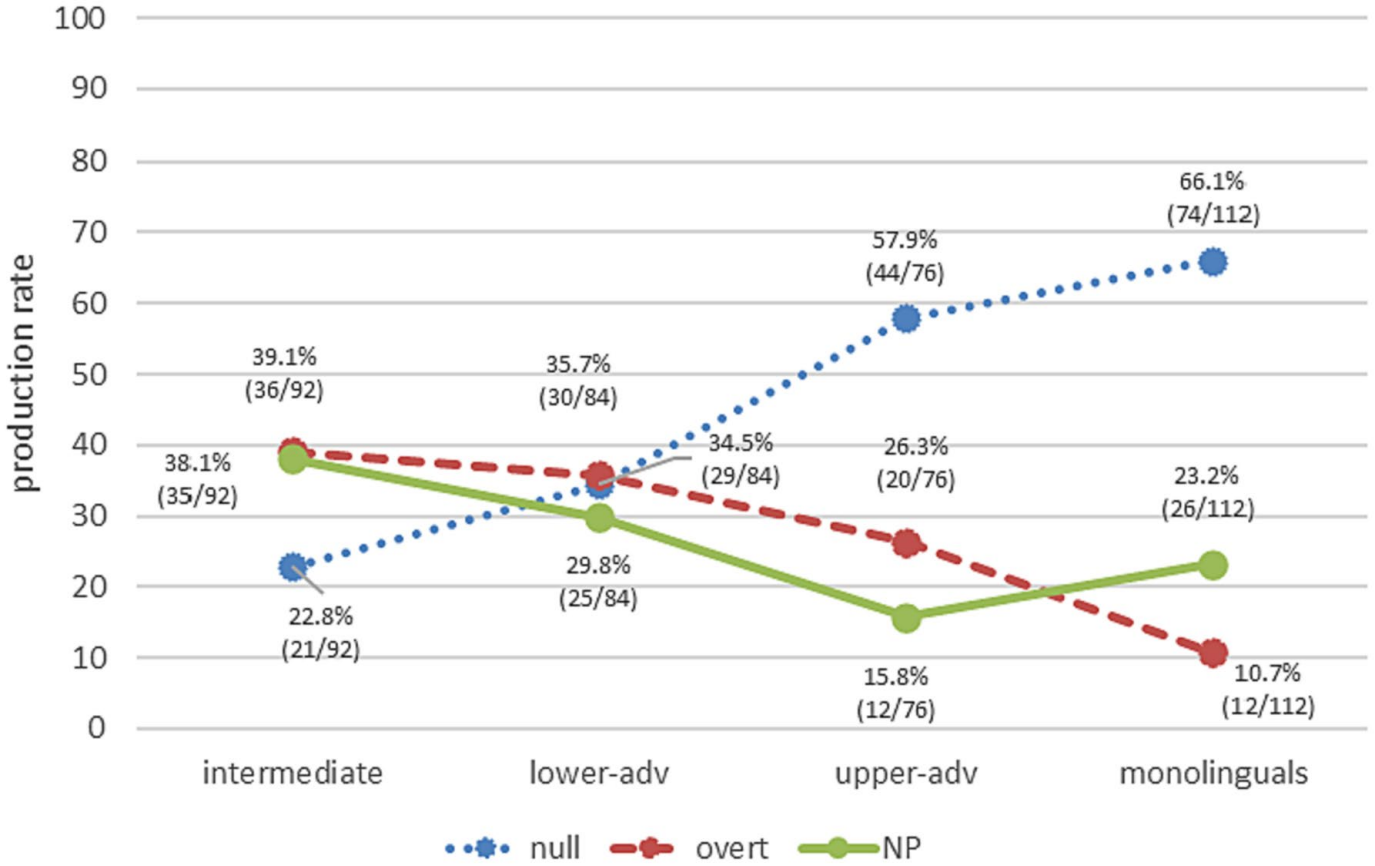
Overall production of REs across groups.

*RQ2* explores the different RE forms in PAS scenarios, independently from the factors that constrain their choice. Spanish monolinguals produced mostly null pronominal subjects (66.1%), followed by NPs (23.2%) and overt pronominal subjects (10.7%) (Figure 4). Learners show a tendency toward the native norm as proficiency increases, yet only upper-advanced leaners (57.9% null, 26.3 overt, 15.8% NP) show a rather similar and non-significant pattern to the Spanish monolinguals (null pronouns: *χ*^2^ = 1.30, *p* = 0.2551, *h* = 0.169; NPs: *χ*^2^ = 1.55, *p* = 0.2135, *h* = 0.188), though a significant difference for overt pronouns (*χ*^2^ = 7.80, *p* = 0.0052, *h* = 0.410). The lower-advanced group shows similar proportions for all three RE forms (34.5% null, 35.7% overt, 29.8% NP), which significantly differ from monolinguals for null (*χ*^2^ = 19.16, *p* < 0.001, *h* = 0.642) and overt (*χ*^2^ = 17.82, *p* < 0.001, *h* = 0.614), but are non-significant for NPs (*χ*^2^ = 1.07, *p* = 0.3012, *h* = 0.149). Intermediates produce mainly overt REs (overt pronouns 39.1%; NPs 38.1%) and some null pronouns (22.8%), with the three RE production rates being significantly different from monolinguals (overt: *χ*^2^ = 22.67, *p* < 0.001, *h* = 0.685; NP: *χ*^2^ = 5.30, *p* = 0.0213, *h* = 0.324; null: *χ*^2^ = 37.96, *p* < 0.001, *h* = 0.902).

These findings support *H2*. Whereas null pronouns are the tendency in Spanish monolinguals and in upper-advanced learners, the rest of learners differ from monolinguals and show more variability in RE forms. Null pronominal subjects are gradually acquired with proficiency level, whereas overt pronouns show the opposite pattern. Crucially, NPs are a frequent RE form to resolve anaphora in PAS scenarios for both learners and monolinguals. We turn next to the division of labor of such RE forms.

### 3.3. *RQ3*/*H3*: division of labor of the different anaphoric forms

**Figure 5:**
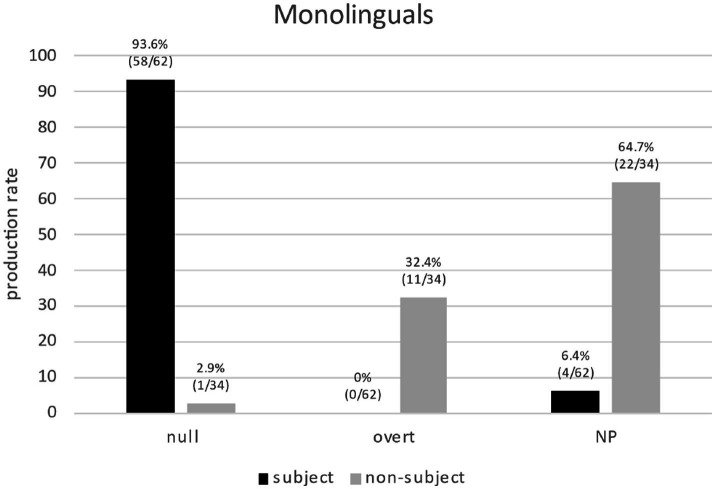
Monolinguals’ production of REs (null/overt/NP) for subject/non-subject antecedents.

First, we focus on Spanish monolinguals’ production to clarify the division of labor in PAS scenarios and to settle the question of whether the alleged flexibility of overt pronouns is more apparent than real. Figure 5 shows a clear bias of null pronouns (93.6%) toward subject antecedents (13), which confirms the PAS and supports most previous research in Spanish. Overt pronouns (32.4%) show a timid bias toward non-subject antecedents, (16b), as previously reported in the literature but, crucially, if we include NPs as a possible RE form, NPs show a strong bias (64.7%) toward non-subject antecedents, (20). Thus, NPs play an important role in PAS scenarios and this could explain the apparently “flexible” bias found for overt pronouns previously reported.

(20) Él_i_ acaba rechazándola_j_ así que **la chica**_**j**_ harta de… [Monolingual: ES_WR_30_3_JVM].

“He_i_ ends up rejecting her_j_, so **the girl**_**j**_, being fed up with…”.

**Figure 6:**
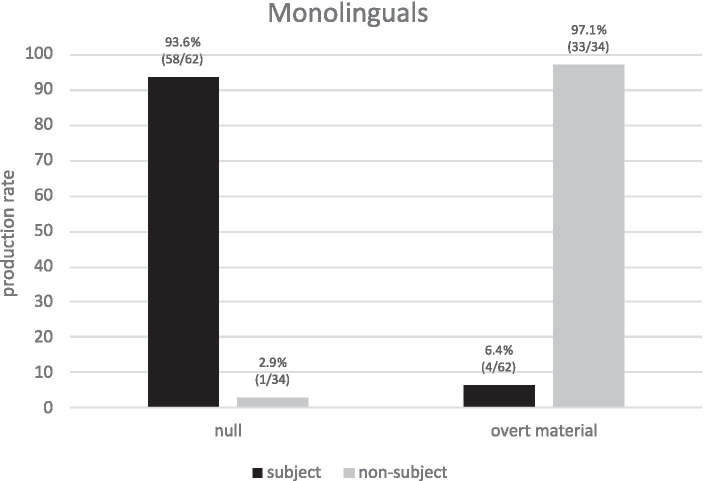
Monolinguals’ production of REs (null vs. overtly realized REs) for subject/non-subject antecedents.

Importantly, if we consider overt and NPs forms together (overtly realized REs), then a neater division of labor shows up (Figure 6): null pronouns are biased toward subject antecedents (93.6%) yet overtly realized REs are biased toward non-subject antecedents (97.1%). Thus, corpus data reveals that the division of labor of AR in native Spanish is more complex and more clear-cut than previously assumed since NPs play a key role. These findings explain the division of labor in native Spanish and therefore settle the dispute on the apparent flexibility of overt pronouns in PAS scenarios.

**Figure 7:**
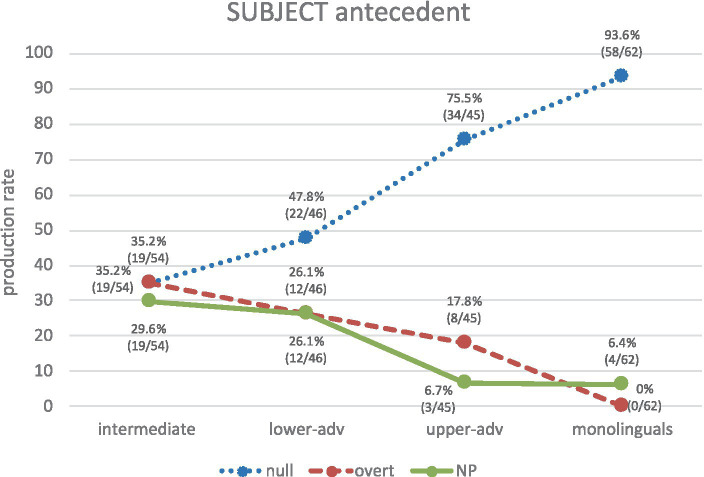
Production of REs for subject antecedents across groups.

Let us now compare learners against monolinguals regarding the production of RE forms for subject vs. non-subject antecedents. As for subject-antecedent biases (Figure 7), Spanish monolinguals show a clear-cut bias as they produce almost exclusively null pronominal subjects (93.6%). Intermediates show equal variability across all three RE forms (null 35.2%, overt 35.2%, NP 29.6%), as illustrated in (21a, b, c) respectively, and their production is significantly different from monolinguals (null: *χ*^2^ = 44.05, *p* < 0.001, *h* = 1.358; overt: *χ*^2^ = 26.09, *p* < 0.001, *h* = 1.270; NP: *χ*^2^ = 10.87, *p* = 0.0010, *h* = 0.638). From lower advanced to upper advanced we can see an increasing trend toward the native norm, particularly for null pronouns (lower advanced: null 47.8%, NP 26.1%, overt 26.1%; upper advanced: null 75.5%, overt 17.8, NP 6.7%), though, crucially, each advanced group significantly differs from the monolingual group: lower advanced vs. monolinguals (null: *χ*^2^ = 28.75, *p* < 0.001, *h* = 1.101; overt: *χ*^2^ = 18.20, *p* < 0.001, *h* = 1.072; NP: *χ*^2^ = 8.07, *p* = 0.0045, *h* = 0.558); upper advanced vs. monolinguals (null: *χ*^2^ = 7.00, *p* = 0.0081, *h* = 0.521; overt: *χ*^2^ = 11.91, *p* = 0.0006, *h* = 0.870; except for NPs, where there are no significant differences *χ*^2^ = 0.00, *p* = 0.9646, *h* = 0.009). In short, intermediates know that a null pronoun can select a subject antecedent, but they equally produce overt pronouns (as in their L1) and NPs. Nearly half of the productions of lower-advanced learners are null pronouns. Upper-advanced learners show native-like discriminations, but their productions are not fully native-like yet.

(21) a. Brooke_i_ despide al maestro_j_ y **Ø**_**i**_ emplea Elle_k_…[Learner: EN_WR_31_21_7_3_DNP].

“Brooke_i_ fires the teacher_j_ and **Ø**_**i**_ employs Elle_k_…”

b. La madre_i_ es sumisa al padre_j_ a través de la película. **Ella**_i_ no ha sabido… [Learner: EN_WR_25_22_17_3_BBB].

“The mother_i_ is submissive to the father_j_ throughout the film. **She**_**i**_ did not know…”

c. Rose_i_ quiere a ve Jack_j_ así que **Rose**_i_ busca a Jack_j_. [Learner: EN_WR_26_18_3_3_BRS].

“Rose_i_ wants to see Jack_j_ so **Rose**_**i**_ looks for Jack_j_.”

**Figure 8:**
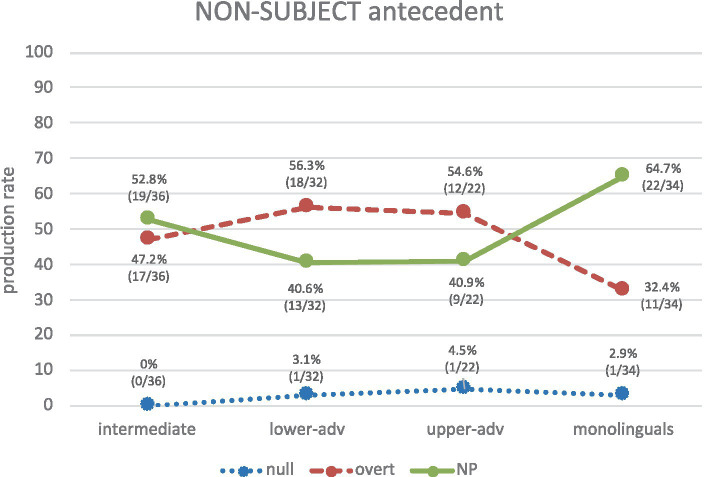
Production of REs for non-subject antecedents across groups.

Consider now non-subject antecedent biases (Figure 8). Spanish monolinguals’ production clearly indicates that NPs (64.7%) (and not overt pronouns) are the privileged RE form to refer to a non-subject antecedent. Crucially, null pronouns are hardly an option for any group, so learners know from the outset that a null pronoun is not an adequate form to refer to a non-subject antecedent. It is therefore remarkable that no null pronouns are used in purely structural PAS configurations. As for learners, overt pronouns and NPs are highly produced, but learners are rather indeterminate about them, particularly intermediates, who show optionality in their production (47.2% overt vs. 52.8% NP), and the two advanced groups, who also show a rather indeterminate pattern where overt pronouns are slightly higher than NPs, as in (22 a, b): lower advanced (56.3% vs. 40.6%), upper advanced (54.6% vs. 40.9%).

(22) a. Bond_i_ encuentra Vesper_j_, y ella_j_ se disculpa. [Learner: EN_WR_36_19_5_3_MWB].

“Bond_i_ finds Vesper_j_ and **she**_**j**_ apologizes.”

b. …ella_i_ escribe algunas cartas a Michael_j_. Pero Michael_j_ no responde. [Learner: EN_WR_38_9_30_3_JG].

“…she_i_ writes some letters to Michael_j_. But Michael_j_ does not reply.”

Figure 8 visually shows that the learners’ pattern is either optional (intermediates) or somewhat opposite to the monolinguals’ (advanced groups). The low frequencies in production in all groups may explain why no significant differences are observed between each of the learner groups and the monolinguals (*p* > 0.05 in all cases, though *p* < 0.50 for each of the advanced groups vs. the monolinguals, which represent marginally non-significant differences).

These findings, taken together, support *H3* since null pronouns show a strong bias toward subject antecedents (with learners showing an increasing sensitivity to this), whereas overt material (i.e., overt pronouns as well as NPs) shows a clear bias toward non-subject antecedents.

### 3.4. *RQ4*/*H4*: the syntax-discourse interface

Recall that, at this point, we need to discriminate between purely structural PAS results (RQ 3, previous section) from purely information status/discursive PAS results (*RQ4*, this section). This will allow us to determine whether the traditional assumption of a correspondence/overlap between syntactic position (subject/non-subject) and information status (topic continuity/shift), as stated in section 1.2, is reflected in production data. Recall that *RQ4* will additionally allow us to check for possible deficits at the syntax-discourse interface, as predicted by the IH.

**Figure 9:**
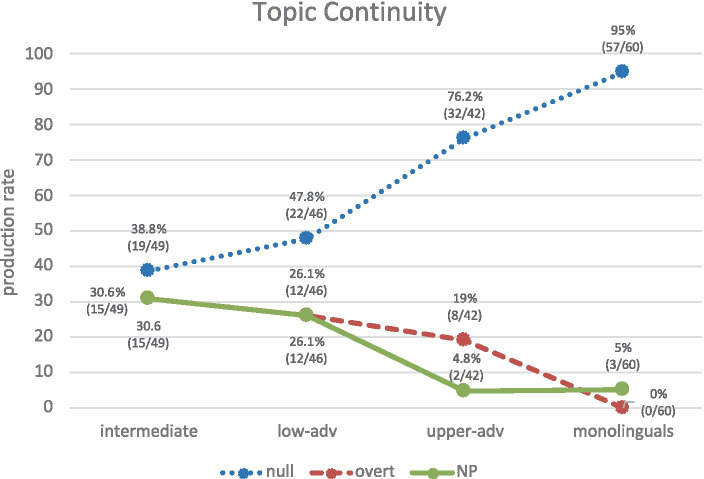
Production of REs in topic-continuity contexts across groups.

Figure 9 shows the use of REs in topic-continuity contexts, where the production of null pronominal subjects is higher for all groups, although the percentages between groups vary considerably. There is a clear increase of nulls from the intermediate to the monolingual group: intermediate (38.8%), lower-adv (47.8%), upper-adv (76.2%), monolingual (95%). If we compare these results with Figure 7, we can observe a similar trend in the results and a similar statistical behavior. In particular, intermediates show again similar variability across all three RE forms (null 38.8%, overt, 30.6%, NP 30.6%), as shown in (23a-c) respectively and their production is significantly different from monolinguals (null: *χ*^2^ = 40.39, *p* < 0.001, *h* = 1.346; overt: *χ*^2^ = 21.30, *p* < 0.001, *h* = 1.173; NP: *χ*^2^ = 12.83, *p* = 0.0003, *h* = 0.772). From lower advanced to upper advanced we can see again an increase toward the native norm, particularly for null pronouns (lower advanced: null 47.8%, NP 26.1%, overt 26.1%; upper advanced: null 76.2%, overt 19%, NP 4.8%), though, once again, each advanced group significantly differs from the monolingual group: lower advanced vs. monolinguals (null: *χ*^2^ = 30.52, *p* < 0.001, *h* = 1.163; overt: *χ*^2^ = 17.65, *p* < 0.001, *h* = 1.072; NP: *χ*^2^ = 9.53, *p* = 0.0020, *h* = 0.621); upper advanced vs. monolinguals (null: *χ*^2^ = 7.86, *p* = 0.0050, *h* = 0.568; overt: *χ*^2^ = 12.40, *p* = 0.0004, *h* = 0.903; except for NPs again, where there are no significant differences (*χ*^2^ = 0.00, *p* = 0.9563, *h* = 0.011).

(23) a. Rose_i_ deja su madre_j_ y Cal_k_ y Ø_i_ va a buscar Jack_l_. [Learner: EN_WR_26_18_3_3_BRS].

“Rose_i_ leaves her mother_j_ and Cal_k_ and **Ø**_**i**_ goes to find Jack_l._”

b. Un día el hombre_i_ estaba sentado en la selva y Ø_i_ vio la dictadora_j_ y después **él**_i_ vio un tigre…[Learner: EN_WR_31_20_Unknown_STS].

“One day the man_i_ was sitting in the jungle and Ø_i_ saw the dictatress_j_ and then **he**_**i**_ saw a tiger…”.

c. … un hombre_i_ muy rico quiere Satine_j_. **El hombre rico**_i_ tiene mas poder que el hombre pobre_k_. [Learner: EN_WR_35_20_10_3_CES].

“A very rich man_i_ loves Satine_j_. **The rich man**_i_ has more power than the poor man_k_.”

**Figure 10:**
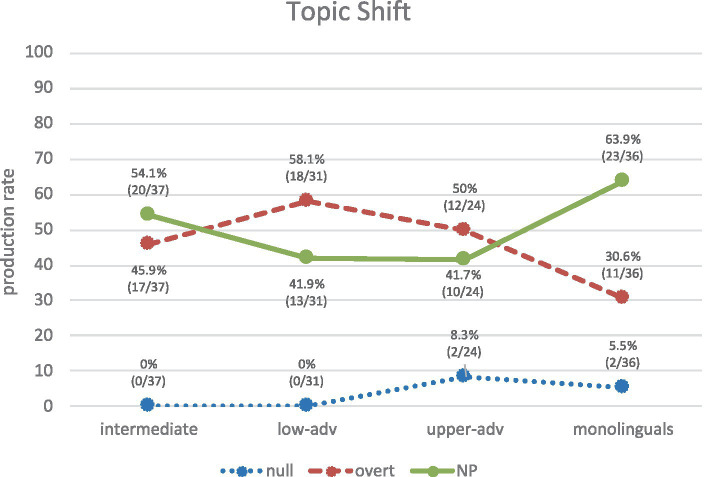
Production of REs in topic-shift contexts across groups.

By contrast, Figure 10 shows the use of REs in topic-shift contexts. Again, these results show a similar trend to those in Figure 8: monolinguals produce mainly NPs (63.9%), followed by overt pronouns (30.6%). Lower-adv and upper-adv learners show a trend that is rather inverse (though less marked) to monolinguals’, by producing overt (58.1, 50%) followed by NPs (41.9, 41.7%), as in (24a, b). Intermediates produce more NPs (54.1%), closely followed by overt (45.9%). Once again, the rather low frequencies in production in all groups may be behind the non-significant differences between each of the learner groups and the monolinguals: non-significant differences (*p* > 0.05) in most contrasts; marginally non-significant differences (0.05 < *p* < 0.10) for NPs in the lower-advanced vs. monolinguals contrast (*χ*^2^ = 3.23) and the upper-advanced vs. monolinguals contrast (*χ*^2^ = 2.87); and only one significant difference for overt pronouns in the lower-advanced vs. monolinguals contrast (*χ*^2^ = 5.13, *p* = 0.0234, *h* = 0.561).

(24) a. …Ben_i_ tenía memorias de su esposa_j_ y su vida con ella_j_. Ella_j_ estaba muy bonita… [Learner: EN_WR_37_18_5_3_JEP].

“…Ben_i_ had memories of his wife_j_ and his life with her_j_. **She**_**j**_ was very pretty…”.

b. Pilar_i_ empieza de desarrollar sus propias opiniones, fuera de su esposo_j_. Su esposo_j_ ha empezado una clase donde Ø_i_ aprende… [Learner: EN_WR_41_19_5_3_AEM].

“Pilar_i_ begins to develop her own opinions, outside her husband_j_. **Her husband**_**j**_ has started a class where Ø_i_ learns…”.

The results in Figures 9, 10 thus show that syntactic position (subject/non-subject) overlaps with information status (topic continuity/shift) in such a way that null pronouns typically mark a continuation of topic of the subject antecedent, whereas overt material (NPs and overt pronouns) typically marks a shift in topic. These results empirically demonstrate that the traditional experimental assumption in section 1.2 is correct in corpus production data.

When it comes the syntax-discourse interface, recall that Sorace’s IH predicts deficits with AR even at advanced levels. This is confirmed in this study, but only partially since our results show that not all syntax-discourse PAS scenarios are equally problematic. In topic-continuity contexts, despite learners’ steady increase of null pronouns, the upper-advanced group (76.2%) still significantly differs from monolinguals (95%), but no significant differences were found in topic-shift scenarios with either NPs or overt pronouns. This differential effect is in line with Lozano’s (2016) Pragmatic Principles Violation Hypothesis (PPHV), originally proposed for general AR in L1 English-L2 Spanish but also confirmed in other scenarios: AR in L1 Greek-L2 Spanish (Lozano, 2018; Margaza and Gavarró, 2022); AR in L1 English-L2 Spanish and L1 Spanish-L2 English (Quesada, 2021); clitic pronouns in L1 English-L2 Spanish (García-Tejada, 2022); and pragmatic implicatures in L1 Chinese-L2 English (Feng, 2022). The PPVH postulates that learners typically obey the pragmatic Principle of Clarity (i.e., they attain native-like knowledge in topic-shift contexts by using full RE forms to avoid ambiguity) but often violate the Principle of Economy (i.e., they produce overt pronouns in topic continuity, which leads to redundancy).

To summarize, the results showed that the choice of REs depends both on (i) the syntactic position of its antecedent (null➔subject vs. NP/overt➔non-subject), and (ii) the information status of its antecedent (null➔topic continuity vs. NP/overt➔topic shift). *H4* is confirmed as there is a correspondence between syntactic position and information structure in PAS. Finally, the PPVH is confirmed since the most advanced L2ers cannot attain full native-like competence in topic-continuity contexts, but they can in topic-shift contexts.

### 3.5. *RQ5*/*H5*: cross-linguistic influence

Recall that a null-subject language like Spanish allows null pronominal subjects in all syntactic configurations (coordination and non-coordination), whereas a non-null subject language like English allows them only in topic continuity *and* coordination. If L1 transfer plays a role in PAS, L1 English-L2 Spanish learners are expected to produce null pronouns mostly in contexts where English allows them.

**Figure 11:**
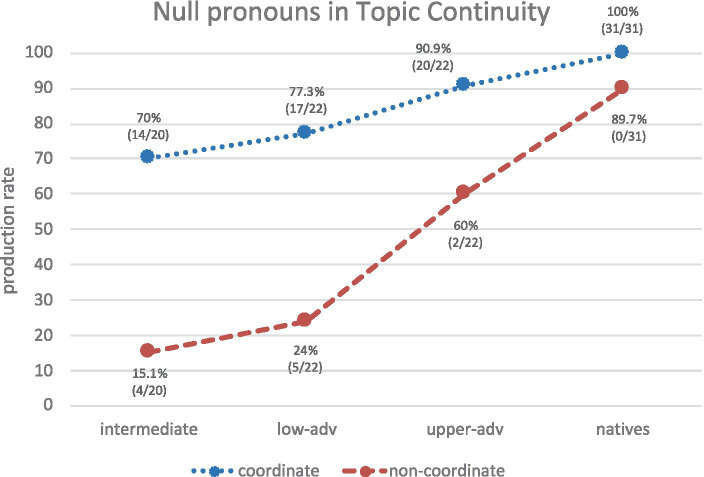
Production of REs in topic continuity and coordinate contexts across groups (only null pronouns plotted).

In topic continuity and coordinate syntactic configurations (*cf.* (16b)), all groups produce mostly null pronominal subjects. Learners show a slight increasing trend toward the native norm, though only the upper-advanced group shows native-like knowledge (Figure 11): intermediates vs. monolinguals (*χ*^2^ = 10.54, *p* = 0.0012, *h* = 1.159); lower-advanced vs. monolinguals (*χ*^2^ = 7.78, *p* = 0.0053, *h* = 0.994); upper-advanced vs. monolinguals (*χ*^2^ = 2.93, *p* = 0.0870 n.s, *h* = 0.613). By contrast, in topic continuity and non-coordinate configurations learners’ production of null subjects (*cf.* (17) and (18)) is much lower and is always significantly different from monolinguals’: intermediates vs. monolinguals (*χ*^2^ = 34.27, *p* < 0.001, *h* = 1.687); lower-advanced vs. monolinguals (*χ*^2^ = 23.97, *p* < 0.001, *h* = 1.463); upper-advanced vs. monolinguals (*χ*^2^ = 5.98, *p* = 0.145, *h* = 0.715). Additional within-group comparisons^13^ show that Spanish monolinguals’ production of null pronouns in topic-continuity coordinate vs. non-coordinate configurations is not significantly different, as expected (*χ*^2^ = 3.38, *p* > 0.05, n.s), whereas learners’ production is significantly different: intermediates (*χ*^2^ = 16.29, *p* < 0.02); lower-advanced (*χ*^2^ = 13.29, *p* < 0.02); upper-advanced (*χ*^2^ = 5.52, *p* < 0.02). Results suggest that learners’ significantly higher use of null pronouns in coordinate than in non-coordinate configurations reflects L1 English influence. Interestingly, learners show a strong gradual trend toward the native norm (intermediate 15.1%, lower-adv 24%, upper-adv 60%), which suggests their sensitivity to the allowability of null pronouns in non-coordinate scenarios increases with proficiency, though their production rates (even at upper-advanced levels) are far from Spanish monolinguals’. This confirms learners’ transfer of null pronouns but an increasing sensitivity to their pragmatics.

### 3.6. *RQ6*: sentential configuration

Table 2 shows that that the production of intersentential configurations is two thirds (or higher) the production of intrasentential configurations for both L2ers and monolinguals, which indicates that the most natural sentential configuration for AR in PAS scenarios is intersentential, either independent sentences as in *[sentence].[sentence]* or coordinate sentences as in *[sentence]&[sentence]*. This clear-cut trend has been rather overlooked in the design of stimuli in previous experimental studies, where intrasentential configurations like *[main [subordinate]]* have been typically the focus of attention. Importantly, only the upper-advanced learners can attain native-like competence as they are not significantly different from Spanish monolinguals (*χ*^2^ = 0.60, *p* = 0.4371, *h* = 0.116), whereas the intermediates (*χ*^2^ = 5.57, *p* = 0.0182, *h* = 0.338), and the lower-advanced learners (*χ*^2^ = 11.31, *p* = 0.0008, *h* = 0.506) significantly differ from monolinguals.

**Table 2:** Syntactic configuration: inter- vs. intra-sentential.

		Intermediate		Lower-adv		Upper-adv		Monolinguals	
INTER-SENT.		79.3% (73/92)		85.7% (72/84)		69.7% (53/76)		64.3% (72/112)	
INTRA-SENT.	Main_subord	20.7% (19/92)	94.74% (18/19)	14.3% (12/84)	83.3% (10/12)	30.3% (23/76)	91.3% (21/23)	35.7% (40/112)	90% (36/40)
	Subord_main		5.26% (1/19)		16.67% (2/12)		8.7% (2/23)		10% (4/40)

Recall that an additional question concerns the order of main and subordinate clauses. Table 2 shows the clausal order for the low-frequency intrasentential configurations: Main-subordinate is overwhelmingly more frequent than subordinate-main for both L2ers and monolinguals. Note that no inferential statistics are performed here due to the low frequencies.

In short, in natural production PAS scenarios are overwhelmingly intersentential and, when they happen to be intra-sentential, the most frequent clausal order is main-subordinate. This is so in native and non-native grammars. These findings provide clear tips for those researchers wishing to design experimental PAS configurations that intend to look as natural as possible.

**Figure 12:**
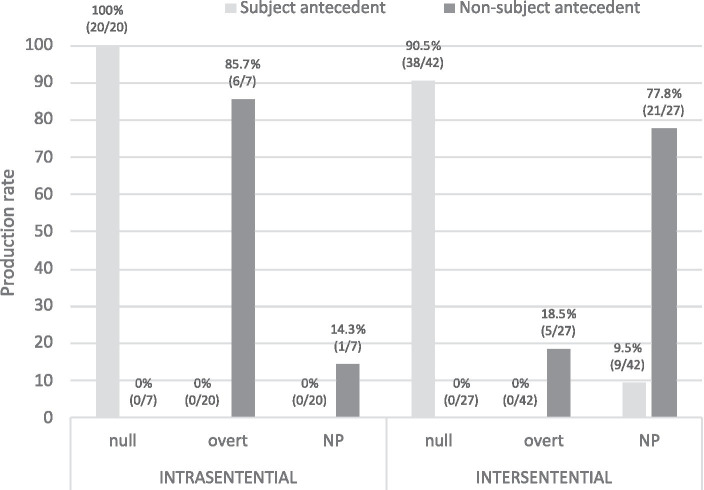
Spanish monolinguals’ production of REs in intrasentential vs. intersentential.

A final consideration is whether the sentential configuration is a factor that modulates the choice of RE in PAS in native Spanish (Figure 12). Null pronominal subjects are clearly biased toward subject antecedents regardless of the type of sentence (100% intrasentential, 90.5% intersentential), whereas an interesting subdivision of labor is observed when the bias is toward non-subject antecedents: overt pronouns in intrasentential (85.7%) but NPs in intersentential (77.8%), as shown in (25a, b). In other words, topic continuity (subject bias) is marked via null pronouns irrespective of the sentential configuration, but topic shift (non-subject bias) is marked via overt pronouns intrasententially yet via NPs intersententially, which is a finding not reported in the previous literature. Sentential configuration is therefore an additional factor that modulates the division of labor of REs in PAS configurations in native Spanish. This issue merits further investigation in future studies containing larger frequencies of learner and native corpus data.

(25) a. Intrasentential: overt pronoun biasing toward a non-subject antecedent:

Marco_i_ está celoso y Ø_i_ no se adapta bien a esta nueva vida de Verónica_j_ cuando **ella**_j_ empieza a tomar a sus amigos como amantes. [Learner: EN_WR_42_21_8_3_LBK].

“Marco_i_ is jealous and Ø_i_ does not adapt well to this new idea of Veronica_j_ when **she**_**j**_ starts taking her friends as lovers.”

b. Intersentential: NP pronoun biasing toward a non-subject antecedent:

Ella_i_ le_j_ tiene mucho cariño, pero Ø_i_ se niega a desmentir sus votos para estar con él_j_. **Nacho**_j_ se deja guiar por un idealismo optimista… [Learner: EN_WR_42_21_10_3_LBK].

“She_i_ is very fond of him_j_, but Ø_i_ refuses to deny her vows to be with him_j_. **Nacho**_**j**_ allows himself to be guided by an optimist idealism…”.

## 4. General discussion and conclusion

*RQ1* called into question the PAS as a prototypical way of resolving anaphora in native (and L2) Spanish. Our corpus results confirmed a low production of PAS compared to other AR configurations in natural language production. So, as we found during the corpus sample selection (section 2.2), it is difficult to find PAS in natural narrative production and, in those narrations that include PAS, their frequency is rather low. Carminati’s (2002) original PAS proposal for Italian has triggered a wealth of experimental studies in many languages and bilingual populations. These studies have blindly tested PAS (and slight variants of it) over and over again but our corpus data show that the PAS is neither a common phenomenon nor prototypical way of resolving anaphora.

Results from *RQ2* confirmed the hypothesis that Spanish native discourse contains mainly null pronominal subjects, while learners’ production is significantly lower. Importantly, two crucial findings for native Spanish PAS were (i) the rather low production of overt pronouns, which contrasts with their importance in experimental studies, and (ii) the high production of NPs as an anaphoric device, an overlooked factor in experimental studies. Learners’ PAS behavior ranged from intermediates’ strong influence from their L1 English (overt pronouns and NPs predominate, with low rates of null pronouns), the indeterminacy of lower advanced learners (production of one third of each RE form), and difficulty to attain native levels by the upper-advanced group since they still produce significantly more overt pronouns than monolinguals, in line with previous corpus research on L2 Spanish dealing with AR in general (Montrul and Rodríguez Louro, 2006; Lozano, 2009, 2016). These findings become more meaningful when we incorporate syntax-discourse factors in PAS, as we will discuss below.

*RQ3* addressed a much-debated topic in the literature on native Spanish: the division of labor of RE forms in PAS. Experimental studies report a clear role for null pronouns (they show a strong subject-antecedent bias), yet overt pronouns show a “flexible” behavior (non-subject- as well as subject-antecedent biases). The corpus data showed a clear division of labor when we consider overtly realized REs together (i.e., overt pronouns and NPs): null pronouns clearly select subject antecedents whereas overt REs clearly select non-subject antecedents. This is quite revealing as NPs were not typically considered in previous experimental PAS studies (except for Gelormini-Lezama and Almor, 2011). The relevance of corpus data then becomes clear as a complementary (and needed) source of evidence for experimental data in the study of bilingualism.

As for learners’ subject antecedents, they start off by showing indeterminacy and no clear PAS strategy in L2 Spanish, but then show a gradual development toward the native norm, but even the upper-advanced group still significantly produces more overt pronouns (and less null pronouns) than monolinguals do to refer to the subject. The results are in line with previous studies regarding development (Jegerski et al., 2011) and native-like knowledge but lack of full native-like attainment at advanced levels (Bel et al., 2016b; Clements and Domínguez, 2017). As for learners’ non-subject antecedents, if we consider overt pronouns and NPs together, the bias is clearer for all groups as overt REs are biased toward non-subject antecedents. So, it seems that the division of labor in learners’ is clearer from early stages for non-subject antecedents than for subject antecedents. This is not surprising as the antecedent bias is somehow related to the information status (i.e., topic continuity/shift) and topic continuity is more problematic than topic shift, as we discuss next.

Results for *RQ4* confirmed the correspondence between information status and syntactic configuration (i.e., null pronouns➔subject antecedent/topic continuity; overt pronouns & NPs➔non-subject antecedent/topic shift). Regarding the deficits at the syntax-discourse interface predicted by the IH (Sorace, 2011), learners showed deficits, but there were differential effects, as predicted by the PPVH (Lozano, 2016): Learners showed native-like behavior in topic-shift, but not in topic-continuity contexts, where even upper-advanced learners redundantly use overt pronouns. In short, learners are more redundant than ambiguous with the PAS.

As for *RQ5*, learners’ lack of native-like attainment with PAS is also motivated by transfer of null pronominal subjects from their L1 in topic continuity and coordination (and not in topic continuity and non-coordination), a fact also reported by Martín-Villena and Lozano (2020) for diverse AR contexts. Curiously, the cross-linguistic effect is milder in the opposite direction (L1 Spanish-L2 English), as reported by Quesada and Lozano (2020), so future research could investigate this asymmetry in a more controlled way, e.g., by keeping the task and the type of AR analysis constant but turning the language pairs (L1 English-L2 Spanish vs. L1 Spanish-L2 English) into a variable. Despite transfer, our results also show acquisition effects since learners gradually increase their production of null pronouns in both contexts as their proficiency increases.

As for *RQ6*, our corpus data showed that 1/3 of PAS configurations were intrasentential, of which over 90% were main-subordinate order. Interestingly, some of the studies reviewed above that investigated intrasentential sentences showed contradictory results depending on the order of presentation: main-subordinate order (Chamorro et al., 2016; Bel et al., 2016a) vs. subordinate-main order (Filiaci, 2010; Filiaci et al., 2014; Keating et al., 2016) (*cf.* online Supplementary material for exact details). Importantly, our corpus findings also show that null pronouns are clearly biased toward subject antecedents regardless of the type of sentential configuration, but for non-subject antecedents the configuration modulates the choice of RE: overt pronouns are biased toward non-subject antecedents intrasententially whereas NPs do so in intersententially.

The current study presents certain limitations. A larger corpus sample would have probably yielded more stable findings but recall our difficulty in finding texts containing enough PAS examples. Additionally, the tasks certainly lead speakers to narrate different films or describe different famous people, which generates a wide and heterogeneous variety of AR scenarios in the texts produced. This could be minimized by using a prompted task (e.g., the narration of a short Charles Chaplin video clip).

Our findings show the relevance of learner corpus research to investigate theoretically-motivated L2 phenomena (Lozano, 2021b). Corpus data have uncovered certain key factors that could be certainly implemented in future experiments. Our research group is currently implementing some of these factors into new experiments (NPs as a form of RE, number of potential antecedents, antecedent-anaphor distance, etc). This is in line with recent claims (Mendikoetxea and Lozano, 2018; Gilquin, 2021) that the triangulation of *experimental* and *corpus* methods leads to a more well-rounded understanding of complex linguistic phenomena in bilingualism and SLA.

## Data availability statement

Publicly available datasets were analyzed in this study. This data can be found at: http://cedel2.learnercorpora.com.

## Ethics statement

The studies involving humans were approved by Comité en investigación Humana (Universidad de Granada), 1794/CEIH/2020. The studies were conducted in accordance with the local legislation and institutional requirements. The participants provided their written informed consent to participate in this study.

## Author contributions

All authors listed have made a substantial, direct, and intellectual contribution to the work and approved it for publication.

## Footnotes

^1^Also known as PAH (Position of Antecedent Hypothesis).

^2^Note that in all the experimental studies under review, the stimuli always contain two potential antecedents (one in subject position, another in non-subject position). The advantage of using corpus data is that in natural production PAS structures typically contain more antecedents and in different syntactic positions (see sections 2.4 and 3.1).

^3^The authors compared discourse-coordinating (*mientras* “while”) vs. -subordinating (*cuando* “when”/*después de que* “after”/*desde que* ‘since’) conjuntions. For brevity, we discuss the discourse-coordination results only.

^4^RT differences = *subject antecedent* (RT of object region + RT of PP region) – *object antecedent* (RT of object region + RT of PP region).

^5^RT differences = *subject antecedent* (RT of main clause) – *object antecedent* (RT of main clause).

^6^The discursive/pragmatic properties of AR are not fully acquired until around 15 years of age (Shin and Smith Cairns, 2012), so evidence from these teenage monolinguals should be taken cautiously.

^7^We incorporate NPs and repeated proper Ns as type of anaphoric form, hence we use the wider term Referential Expressions (REs) to include all forms (overt/null pronouns, NPs, repeated Ns), instead of the more restrictive term anaphoric forms.

^8^http://learnercorpora.com

^9^Only monolinguals from Spain were chosen since in certain varieties (Mexican, Caribbean, Puerto Rican), overt pronouns mark topic continuity (Flores-Ferrán, 2004).

^10^http://www.corpustool.com

^11^The original tagging scheme included a richer tagset with more tags that are not analyzed in this study due to space limitations –see Quesada (2021) for details.

^12^After each corpus example, we provide in square brackets the filename from the CEDEL2 corpus (http://cedel2.learnercorpora.com).

^13^The latest release of UAM Corpus Tool (version 6.2j, February 2023) does not allow complex within-group comparisons, so we used an earlier release (version 3.3x, August 2021) to do the analysis, though note that version 3.3x reports *p* value ranges (non significant *p* > 0.05, significant *p* < 0.05, highly significant *p* < 0.02) and does not report effect size.
