# Supplementary material for: What corpus data reveal about the Position of Antecedent Strategy: anaphora resolution in Spanish monolinguals and L1 English-L2 Spanish bilinguals
Source: Front Psychol. 2023 Nov 9;14:1246710. doi: 10.3389/fpsyg.2023.1246710 (PMC10666169; doi:10.3389/fpsyg.2023.1246710)
Supplement: Supplementary file 1 [file Data_Sheet_1.docx]

Online supplementary material

Table 1: PAS in native and L2 Spanish (offline experiments)

| **Study** | **Sample** | **PAS configuration** | **Method** | **Main findings** |
| --- | --- | --- | --- | --- |
| Alonso-Ovalle et al (2002) | Natives: 50 university students (Madrid) | **Intersentential:**  *Juan pegó a Pedro. Ø/Él está enfadado.* | Offline experiment: Sentence interpretation task. | •Ø🡪Subject 🗹  •Overt🡪Subject/Object 🗷 |
| Bel, García-Alcaraz & Rosado (2016) | Natives: 34 Spanish/Catalan university speakers (Barcelona) | **Intrasentential:**  **Main-subordinate:**  *María sorprendió a Raquel mientras Ø/ella se casaba.*  **Subordinate-Main:**  *Mientras Pilar servía a Rebeca, Ø/ella tiró la bebida.* | Offline experiment: Acceptability judgement task with continuation sentence. | All clausal orders:  •Ø🡪Subject 🗹  •Overt🡪Object 🗹  Main-subordinate:  •Ø🡪Object 🗷  •Overt🡪Object 🗹  Subordinate-main:  •Ø🡪Subject 🗹  •Overt🡪Object 🗹 |
| Bel & García-Alcaraz (2015) | Natives: 34 Spanish/Catalan university speakers (Barcelona) | (as the cell above) | (as cell above) | (as cell above) |
|  | Learners: L1 Moroccan Arabic – L2 Spanish (intermediates) | (as the cell above) | (as cell above) | Main-subordinate:  •Ø🡪Subject 🗹  •Overt🡪Object 🗹  Subordinate-main:  •Ø🡪Subject 🗹  •Overt🡪Object 🗹 |
| Jegerski, VanPatten & Keating (2011) | Natives: 26 from Spain and Latin America, university students and professionals studying/working in a US university. | **Intrasentential (Main-subordinate):**  **Discourse coordination:**  *Marta le escribía frecuentemente a Lorena cuando Ø/ella estaba en los Estados Unidos.*  **Discourse subordination:**  *El niño vio a su hermano mientras Ø/él jugaba en el jardín.* | Offline experiment: Sentence Interpretation Task | Discourse coordination:  •Ø🡪Subject 🗹  •Overt🡪Subject/Object 🗷  Discourse subordination:  •Ø🡪Subject 🗹  •Overt🡪Subject/Object 🗷 |
|  | Learners: L1 English-L2 Spanish (intermediate, advanced) learners/professors of Spanish in an US university | (as the cell above) | (as cell above) | Intermediates:  Discourse coordination:  •Ø🡪Subject 🗹  •Overt🡪 Subject 🗷  Discourse subordination:  •Ø🡪 Subject/Object 🗷  •Overt🡪Subject/Object 🗷  Advanced:  Discourse coordination:  •Ø🡪 Subject 🗹  •Overt🡪Subject/Object 🗷  Discourse subordination:  •Ø🡪 Subject/Object 🗷  •Overt🡪Subject/Object 🗷 |
| Keating, VanPatten & Jegerski (2011) | Natives: 19 (same profile as their first study above) | (as the cell above) | (as their first study above) | •Ø🡪Subject 🗹  •Overt🡪Subject/Object 🗷 |
|  | Learners: L1 English-L2 Spanish (advanced): same profile as their first study above | (as the cell above) | (as cell above) | •Ø🡪Subject(/Object) 🗷  •Overt🡪Subject/Object 🗷 |
| Clements & Domínguez (2017a) | Natives: Peninsular Spanish (N=14) and Mexican variety (N=2) | **Intrasentential (Main-subordinate):**  *La mujer empuja a la niña en el columpio mientras Ø/ella se come un helado.* | Picture Verification Task (PVT) | •Ø🡪Subject 🗹  •Overt🡪Object 🗹 |
|  | Learners: L1 English-L2 Spanish (advanced): final-year undergraduates in British university | (as the cell above) | (as cell above) | •Ø🡪Subject 🗹  •Overt🡪Object 🗹 |

Notes:

1. Shaded rows represent group contrasts: light grey (Spanish natives) vs. dark grey (L2 Spanish learners).

3. A ticked box (🗹) indicates that the findings corroborate Carminatti’s original PAS prediction. A crossed box (🗷) indicates otherwise.

Table 2: PAS in native and L2 Spanish (online experiments)

| **Study** | **Sample** | **PAS configuration** | **Method** | **Main findings** |
| --- | --- | --- | --- | --- |
| Gelormini & Lezama (2011) | Natives: 45 Buenos Aires university students (River Plate Argentinian Spanish) | **Intersentential:**  •*Subject biased:*  Juan se encontró con María. Juan_i_/Él_i_/Ø_i_ la_j_ vio triste.  •*Object biased:*  María_i_ se encontró con Juan_j_. Juan_j_/Él_j_/Ø_j_ la_i_ vio triste. | Self-Paced Reading Task (sentence-by-sentence presentation) | •Ø faster to Subject than Object 🗹  •Overt faster to Object than Subject 🗹  •Repeated N equally fast to Subject and Object |
| Bel et al. (2016) | Natives: 34 Spanish/Catalan university speakers (Barcelona) | **Intrasentential (main-sub):**  •*Subject biased:*  El músico_i_ saluda al bombero_j_ mientras Ø_i_/él_i_ lleva un violín en la mochila.  •*Object biased:*  El músico_i_ saluda al bombero_j_ mientras Ø_j_/él_j_ lleva un casco en la mochila. | Self-paced Reading Task (word-by-word, non-cumulative presentation) | •Ø faster to Subject than Object 🗹  •Overt faster to Object than Subject 🗹  (but differences depend on the critical region under analysis: NP object vs. PP) |
|  | Learners: 82 L1 English-L2 Spanish, 68 L1 Arabic-L2 Spanish, at 3 proficiency levels (intermediate, upper-intermediate, high) | (as the cell above) | (as cell above) | Only the high-level learners:  •Ø faster to Subject than Object 🗹  •Overt faster to Object than Subject 🗹 |
| Filiaci (2010) | Natives: 32 Spanish students enrolled at the University of Edinburgh (average 2.4 months of residence, SD: 4.3 months) | **Intrasentential (sub-main):**  •*Subject biased:*  Cuando Ana_i_ visitó a María_j_ en en el hospital, Ø_i_/ella_i_ le llevó un ramo de rosas.  •*Object biased:*  Cuando Ana_i_ visitó a María_j_ en el hospital, Ø_j_/ella_j_ ya estaba fuera de peligro. | Self-paced Reading Task (clause-by-clause presentation) | •Ø faster to Subject than Object 🗹  •Overt equally faster to Object than Subject 🗷 |
| Filiaci et al. (2014), experiment 1 | (as the cell above) | (as the cell above) | (as the cell above) | (as the cell above) |
| Filiaci et al. (2014), experiment 2 | Natives: 32 monolingual speakers undergraduates at University of La Laguna, Canary Islands (Spain) | (as the cell above) | Self-paced Reading Task (phrase-by-phrase presentation) | (as the cell above) |
| Keating et al. (2016) | Natives: 45 monolingual speakers undergraduates at the University of Guanajuato (Mexico) | •*Subject biased:*  Después de que el sospechoso_i_ habló con el policía_j_, Ø_i_/él_i_ admitió su culpabilidad  •*Object biased:*  Después de que el policía_i_ habló con el sospechoso_j_, Ø_j_/él_j_ admitió su culpabilidad | Self-paced Reading Task (clause-by-clause presentation) | •Ø faster to Subject than Object 🗹  •Overt faster to Object than Subject 🗹 |
